# The goal of explaining black boxes in EEG seizure prediction is not to explain models' decisions

**DOI:** 10.1002/epi4.12748

**Published:** 2023-04-27

**Authors:** Mauro F. Pinto, Joana Batista, Adriana Leal, Fábio Lopes, Ana Oliveira, António Dourado, Sulaiman I. Abuhaiba, Francisco Sales, Pedro Martins, César A. Teixeira

**Affiliations:** ^1^ Department of Informatics Engineering CISUC Univ Coimbra Coimbra Portugal; ^2^ Epilepsy Center, Department Neurosurgery, Medical Center ‐ University of Freiburg Faculty of Medicine University of Freiburg Freiburg Germany; ^3^ Refractory Epilepsy Reference Centre Centro Hospitalar e Universitário de Coimbra, EPE Coimbra Portugal; ^4^ CIBIT Coimbra Institute for Biomedical Imaging and Translational Research Coimbra Portugal

**Keywords:** drug‐resistant epilepsy, EEG, explainability, machine learning, seizure prediction

## Abstract

Many state‐of‐the‐art methods for seizure prediction, using the electroencephalogram, are based on machine learning models that are black boxes, weakening the trust of clinicians in them for high‐risk decisions. Seizure prediction concerns a multidimensional time‐series problem that performs continuous sliding window analysis and classification. In this work, we make a critical review of which explanations increase trust in models' decisions for predicting seizures. We developed three machine learning methodologies to explore their explainability potential. These contain different levels of model transparency: a logistic regression, an ensemble of 15 support vector machines, and an ensemble of three convolutional neural networks. For each methodology, we evaluated quasi‐prospectively the performance in 40 patients (testing data comprised 2055 hours and 104 seizures). We selected patients with good and poor performance to explain the models' decisions. Then, with grounded theory, we evaluated how these explanations helped specialists (data scientists and clinicians working in epilepsy) to understand the obtained model dynamics. We obtained four lessons for better communication between data scientists and clinicians. We found that the goal of explainability is not to explain the system's decisions but to improve the system itself. Model transparency is not the most significant factor in explaining a model decision for seizure prediction. Even when using intuitive and state‐of‐the‐art features, it is hard to understand brain dynamics and their relationship with the developed models. We achieve an increase in understanding by developing, in parallel, several systems that explicitly deal with signal dynamics changes that help develop a complete problem formulation.

**Key Points**

- Authors develop ways to explain their models, but they do not evaluate how these explanations help specialists complete their tasks.
- Explanations are insufficient to understand brain dynamics, even when using interpretable models with few but widely used features.
- When there is no solid physiological grounding, simply explaining models will not provide us with any advance in understanding the problem.
- Model explanations are not meant to explain decisions but to set the ground for basing model development on an iterative refutation strategy.

## 1 INTRODUCTION

While medication has a success rate of 70% in achieving seizure control, patients with drug‐resistant epilepsy (DRE) require other strategies, such as seizure prediction, to improve their lives.^1^, ^2^, ^3^, ^4^ By analyzing the electroencephalogram (EEG) signal in real‐time, the design of a warning device that timely anticipates seizures may allow the patient to take action for seizure suppression (administering rescue medication) or to minimize its effects (taking preventive measures against accidents). It may also be possible to integrate a prediction model in a closed‐loop system that automatically performs neuromodulation to suppress seizures.^5^, ^6^, ^7^, ^8^

Seizure prediction encompasses the development of machine learning (ML) models using multidimensional time‐series data, often resulting in black‐box models. The absence of explanations for black‐box models' decisions, especially when they fail, makes researchers question and mistrust their use.^7^ If one tries to explain a model's decision, particularly its failures, it still might convince clinicians.^9^, ^10^

Recently, ML interpretability and explainability^11^, ^12^ gained importance. Concerning interpretability, authors have developed simple models with few features, including sparse linear models^13^, ^14^, ^15^ and low‐depth decision trees.^16^, ^17^ With the advent of *big data* and the increase in computational power, more complex models may now lead to better performances. ML explainability arose as a need to retrieve knowledge from such models.^9^, ^18^ However, none of these studies formally evaluates how these explanations helped specialists complete their tasks. Explanations have a sociological component^9^, ^10^ and must be seen as an exchange of beliefs that help to answer a “why” question when one can no longer keep asking why.^9^, ^12^, ^19^

In this paper, to evaluate and understand what may be the critical model explanations for EEG seizure prediction, we developed and quasi‐prospectively evaluated three ML solutions with different levels of complexity. Then, we selected patients to construct different explanations for their model decisions. We presented the explanations to ML experts and clinicians.

Results showed that current explanations methods are insufficient to understand brain dynamics, even when using interpretable models with few widely used features. As clinicians cannot detect pre‐seizure patterns several minutes before seizures, the goal of explainability is not to explain model decisions but to help improve the system. Explainability allows researchers to design hypotheses regarding physiology and model behavior that helps develop a complete problem formulation. Even when pre‐seizure underlying mechanisms are unknown, we obtain more trust in the models when we test the developed hypotheses, and they still stand.

## 2 MATERIALS AND METHODS

Our methodology comprises three main steps: (i) developing ML methodologies, (ii) developing explanations, and (iii) evaluating explanations. Summarily, we developed three ML pipelines with different degrees of transparency. Then, we selected patients with high and low performances to develop explanations for their models and decisions. Interviews were performed by showing the developed explanations and asking open‐ended questions to scientists working in healthcare and clinicians (neurologists and EEG technicians) working in an epilepsy refractory center. We analyzed the interviews using grounded theory (GT), a qualitative research tool that allowed us to retrieve emergent topics and ideas vital to understanding model explanations and their significance in EEG seizure prediction research. We summarized our findings in four lessons by interpreting the emerged themes and ideas.

### 2.1 Dataset

From the EPILEPSIAE database,^20^ we selected 40 patients with DRE (23 males and 17 females, aged 39.42 ± 15.87) from the Universitätsklinikum Freiburg in Germany. This dataset contains 224 seizures (120 for training and 104 for testing), 3254 hours of training data (≈4.52 months), and 1402 hours of testing data (≈1.95 months). Our patient selection criteria were as follows: (i) patients with temporal lobe epilepsy, as it concerns the most common type of focal epilepsy; (ii) a minimum of four recorded seizures separated by periods of at least 4 h30; and (iii) EEG scalp recorded with a sampling frequency of 256 Hz. Electrodes were placed according to the 10–20 system. The data were collected while patients were in the clinic for presurgical monitoring. The ethical committee approved the use of this data for research purposes of the three hospitals involved in the database development (Ethik‐Kommission der Albert‐Ludwigs‐Universität, Freiburg; Comité consultatif sur le traitement de l'information en matière de recherche dans le domaine de la santé, Pitié‐ Salpêtrière University Hospital; and Ethics Committee of the Centro Hospitalar e Universitário de Coimbra).

All methods were performed following the relevant guidelines and regulations. Informed written patient consent from all subjects and/or their legal guardian(s) was obtained to participate. See Data S1 for detailed information on the selected patients.

### 2.2 Developing ML methodologies

**FIGURE 1:**
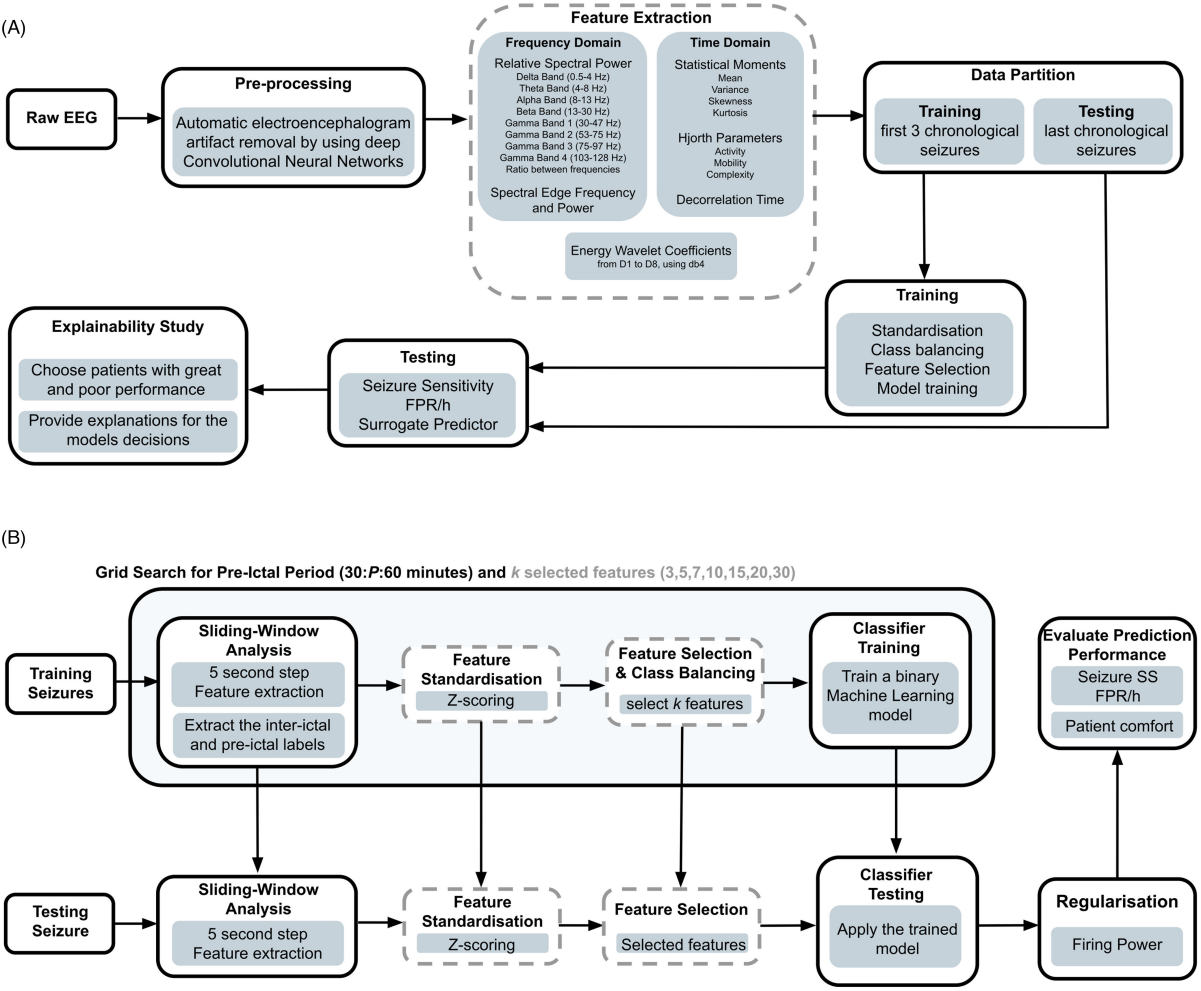
General overview of this study's framework applied in the three methodologies. In (A), we present the general strategy for the entire study. In (B), we provide more details on the ML pipeline, namely the training and testing phases. Steps represented by gray dashed bordered boxes and gray text (feature extraction, selection, and standardization) are performed only for the methodologies that include the SVM and the logistic regression models.

We developed three methodologies using the rationale from Figure 1: a deterministic logistic regression, a voting system of 15 support vector machines (SVMs), and another of three convolutional neural networks (CNN). We preprocessed the raw EEG by using a CNN developed with EPILEPSIAE database^21^ that mimics experts' manual behavior. Then, we extracted univariate linear features from time and frequency domains within 5‐second sliding windows without overlap. For the frequency domain, we extracted the relative spectral power of bands (delta (0.5‐4 Hz), theta (4‐8 Hz), beta (8‐13 Hz), alpha (13‐30 Hz), gamma band 1 (30‐47 Hz), gamma band 2 (53‐75 Hz), gamma band 3 (75‐97 Hz), and gamma band 4 (103‐128 Hz)), the ratio between these bands, spectral edge frequency, and power at 50%. For the time domain, we extracted the four statistical moments (mean, variance, skewness, and kurtosis), Hjorth parameters (activity, mobility, and complexity), and decorrelation time. We also extracted the energy of the wavelet coefficients (D1‐D8, with db4 mother wavelet). See Data S1 for more details on the features, including their expected added value.

All methodologies were patient‐specific. We selected each patient's first three chronological seizures for training (preictal period estimation, standardization, class balancing, feature selection, and model training) and the remaining ones for testing (Figure 1). We used a grid search to obtain the preictal period (30–60 minutes in steps of 10).^5^, ^22^ The preictal period concerns a predefined period before each seizure, where we assumed a pre‐seizure state. We considered the remaining time, named interictal, free of pre‐seizure activity. Each trained classifier learned to distinguish preictal samples from interictal ones. The most straightforward methodology uses the logistic regression, where we balanced the classes' weight in inverse proportion to their frequency. The other two are stochastic and use a voting system. We balanced these by randomly selecting samples from equally spaced segments over the signal to get a representative set of the interictal period. Before training the SVMs, we used a stochastic forest of trees for feature selection.^23^ The CNNs used 5‐second preprocessed windows as input.

For testing seizures, we applied the Firing Power^24^ to smooth the model predictions over time, where we reasonably defined an alarm threshold to 0.7.^13^, ^14^ After each alarm, we applied a refractory period of equal length to the preictal period. We evaluated performance^25^ by calculating seizure sensitivity (SS) and false‐positive rate per hour (FPR/h) and performing a surrogate analysis.^26^ SS is the ratio of correctly predicted seizures. FPR/h is the number of false alarms divided by the interictal period duration (we excluded alarms' refractory period). By randomly shifting seizure onset times (within each seizure data) through Monte Carlo simulations, the surrogate analysis informs that a model performs above chance when its performance is higher than the surrogate one with statistical significance. This test is made under the null hypothesis that each model's performance is not superior to the chance level. We used a seizure prediction horizon^25^ of 10 minutes to allow patient intervention. We used MATLAB R2018b for feature extraction. For the remaining steps, we used Spyder 4.0.1 and Python 3.7. All ML functions are from scikit‐learn. For the CNNs, we used TensorFlow and Keras libraries. See Data S1 for complete details on all pipelines, including CNNs' architecture.

### 2.3 Developing explanations

**FIGURE 2:**
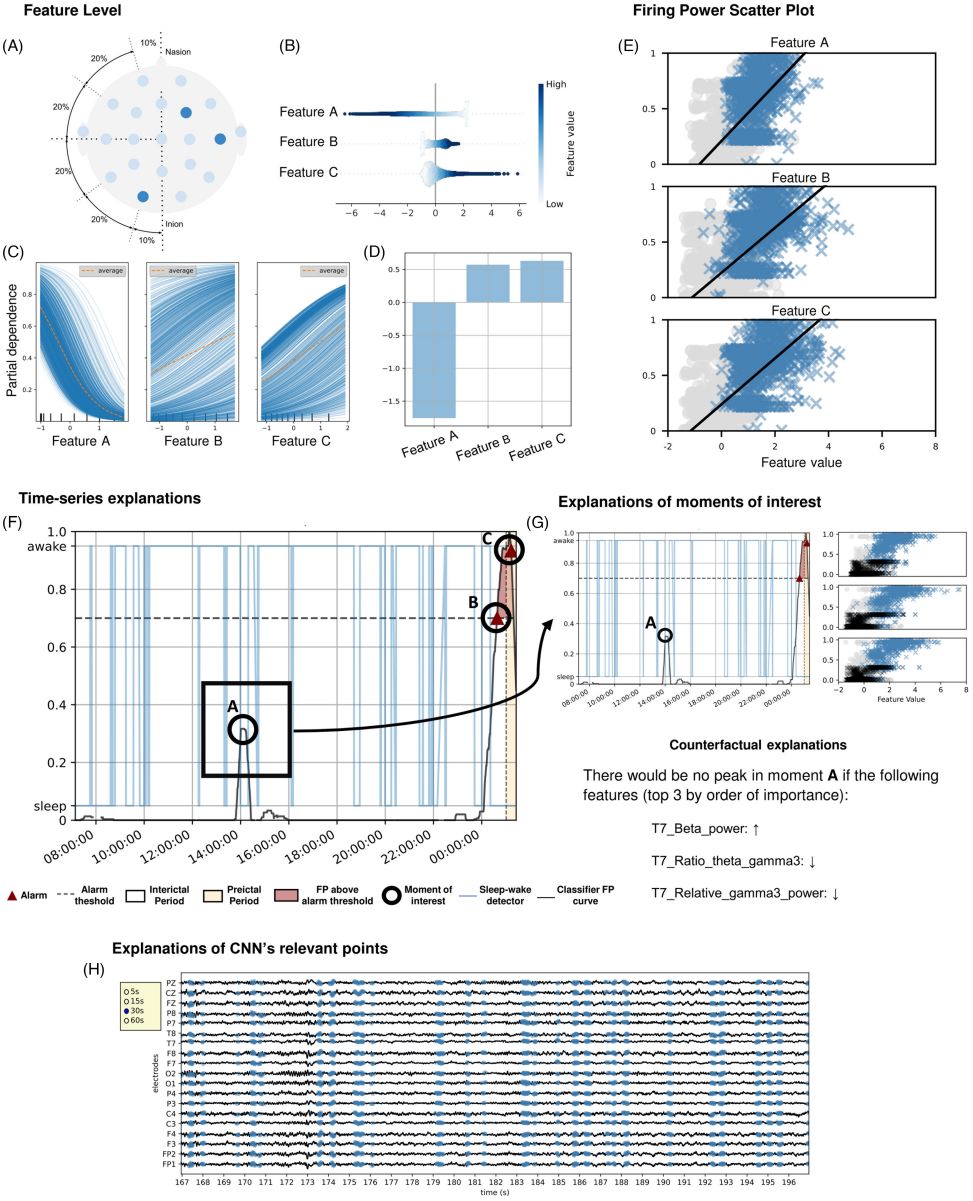
Developed explanations for each patient. We show the selected features and discuss them, namely their expected added value and brain localization. We also discussed anatomical/topological aspects (A). We present each feature's SHAP value (B), ICE, and PDP (C). We also show the regression coefficients (D) for the logistic regression model. We also show Firing Power scatter plots (E), where the *y*‐axis represents a seizure probability occurrence (Firing Power) and the *x*‐axis the feature value. The preictal samples are in blue, and the interictal ones are in gray. The Firing Power plot over the signal (F). This plot also shows the sleep/awake patient state and raised alarms. From all signals, we choose moments that deserve more profound attention, mostly false alarms and seizures that we failed to predict. We then analyze these moments (G) by inspecting the correspondent points on our Firing Power scatter plots and developing counterfactual explanations. When using the CNN, in points of interest, we can also use LIME to inspect the points that led the network to classify the signal as preictal (H).

After assessing performance, we selected patients with: (i) high SS and low FPR/h, (ii) high SS and high FPR/h, (iii) low SS and high FPR/h, and (iv) low SS and low FPR/h. We believe these represent the whole dataset. By only analyzing a reduced number of patients, we could conduct interviews and deepen explanations. Figure 2 depicts an overview of all developed graphical explanations, presented in the interviews.

We ended up focusing on the time‐series results and analyzing the features' expected behavior along with the anatomical/topological aspects. Concerning the time series, we plotted the Firing Power along with a sleep/awake detector,^27^ interictal and preictal periods, raised alarms, and time stamps (Figure 2f). For moments we considered to be of interest (Figure 2g), we plotted the respective points over the Firing Power scatter plot and provided counterfactual explanations. Counterfactual explanations describe a causal situation in the form of “if X had not occurred, Y would not have occurred.”^9^ We computed these explanations by finding the slightest change in each feature that modified the prediction.

For the CNN, we used local interpretable model‐agnostic explanations (LIME)^28^ to show the points in the EEG time series that made the neural network classify segments as preictal (Figure 2h). We only showed these explanations to clinicians since EEG expert knowledge was required. Although clinicians cannot identify brain patterns that lead to seizures several minutes beforehand, we assumed they might provide a physiological interpretation of the algorithm behavior, which would correlate with pre‐seizure brain dynamics.

Other explanations were developed but were not considered relevant for this paper findings. See Data S1 for a description of these. We developed all explanations with Spyder 4.0.1 and Python 3.7, using scikit learn, shap, and lime packages.

### 2.4 Evaluating explanations

We presented the developed explanations during interviews while asking open‐ended questions. These were shown first to 10 data scientists to guarantee that any technical question was addressed. With their feedback, we redefined and presented the set of explanations to 10 clinicians. The interviews were audio recorded. The interview script is in Data S1. The presentation slides of the interviews are present on this paper's Github. After transcribing and anonymizing the interview answers into text, we deleted the audio files. All the interviewees agreed to be recorded. We analyzed the transcribed interviews using GT, an inductive process broadly used in qualitative methods for social sciences.^29^ With GT, the paradigm differs from quantitative methods: Researchers develop a theory from empirical data rather than confirming a hypothesis. GT is an iterative codification and identification of emerging themes from collected data.^30^ We did not use GT to search traditional themes but to understand how we can provide convincing and adequate explanations to experts. We summarized our findings in lessons to best convey our findings.

Despite 10 interviewees for each group (data scientists and clinicians) appear to be small, GT‐based strategies stop when reaching saturation or, in other words, when no more emerging themes and relations can be found.^31^

## 3 RESULTS

In Table 1, we present the seizure prediction results for the entire set of patients and for patients that we selected to develop explanations. We present here the four lessons, where we focus in our most important one: Explainability's goal is not to explain the system's decisions but to improve the system itself. See Data S1 for the complete seizure prediction results and more details about the lessons.

**TABLE 1:** Overall prediction results for the three ML pipelines. The patients that we selected for developing explanations are also present.

Model	Patient	SS (0–1)	FPR/h	Above chance
Logistic regression	Overall	0.13 ± 0.26	0.40 ± 0.46	5 in 40 (0.125)
	8902	1.00	0.11	Yes
	93 402	1.00	0.50	Yes
	101 702	0.00	0.71	No
	402	0.00	0.00	No
Ensemble of 15 SVMs	Overall	0.17 ± 0.28	0.87 ± 1.11	7 in 40 (0.175)
	53 402	1.00	0.22	Yes
	59 102	0.00	0.52	No
	46 702	0.00	0.00	No
Ensemble of 3 CNNs	Overall	0.04 ± 0.10	0.18 ± 0.26	3 in 40 (0.075)
	8902	0.50	0.00	Yes
	23 902	0.00	1.65	No
	32 702	0.00	0.00	No

### 3.1 Discussing features is important

Discussing about the extracted and selected features occupied a large part of the interviews' time. Several data scientists asked for more clinical knowledge concerning these features. Also, many wanted to visualize the time plots of some features, particularly spectral bands' relative powers. Whenever possible, we advise the use of spectral‐band features.

**FIGURE 3:**
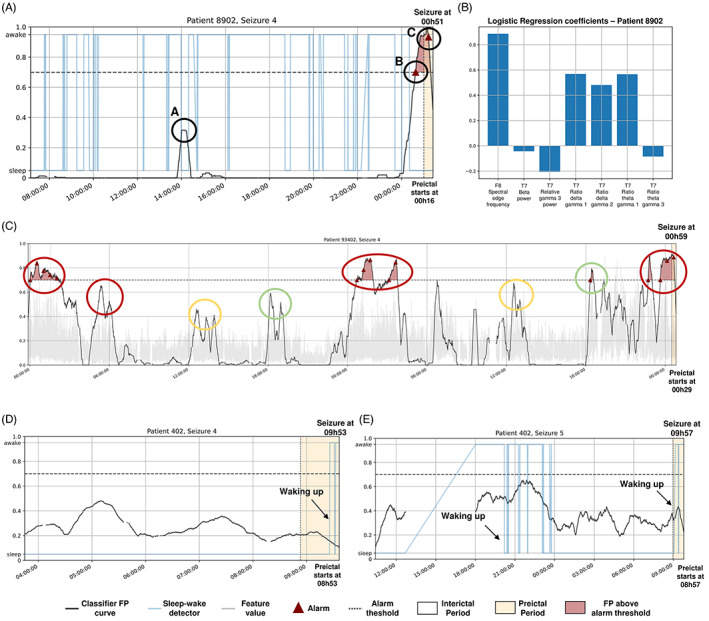
Examples of explanations for the logistic regression model. The time‐series explanation (A) and the corresponding feature regression coefficients (B) are presented for the seizure #4 of patient 8902. The Firing Power and one feature (wavelet energy of the decomposition level 5 from electrode O5) are plotted over time for seizure #4 of patient 93 402 (c). By inspecting each feature individually, very straightforward relations were observed between the models' decisions and the corresponding feature. Each colored circles represent similar signal activity captured at the same time of day (different colors corresponding to repeating patterns showing at different times of the 24‐h day), possibly evidencing the influence of circadian rhythms. Time‐series explanations are also presented when no alarm was raised, for seizures #4 (d) and #5 (e) of patient 402.

Figure 3b depicts the importance of discussing features. It shows the selected features and their influence on patient 8902 for the logistic regression model. As one can see, gamma band‐related measures appeared in five out of the seven selected features. Clinicians found this suspicious as scalp EEG does not fully capture gamma rhythms, and thus, this predominance of gamma features might be explained by the presence of artifacts. We discussed the following hypothesis: *patient 8902 presents movement jerks caused by EEG pre‐seizure dynamics*. This was just a hypothesis as we did not have the video EEG. In this section, we later explain how we tested this hypothesis without accessing video EEG.

Curiously, topological/anatomical explanations of the chosen electrodes were not particularly fundamental compared with features. When the selected electrodes concerned the focus regions, this made sense and provided higher trust. However, when the selected electrodes were far from the focus region, it could be due to the network theory, which proposes that even focal seizures may arise from abnormal activity resulting from a large‐scale functional network^5^, ^6^, ^14^ that spans across lobes and hemispheres. Thus, it was never possible to reach any conclusion without collecting more data and testing.

### 3.2 Time plots are the most intuitive explanations

Time plots are great for raising questions. By inspecting the time plots from Figure 3, some patterns were observed, allowing to present hypotheses to clinicians (as explanations).

For example, we may try to explain the false alarm (moment B) after midnight before seizure #4 from patient 8902 by inspecting (Figure 3a). When visualizing the Firing Power from 8 am until midnight, despite presenting a relatively small peak of 0.40 (moment A), we verified that our system was far from raising an alarm. Afterward, the Firing Power presented a monotonically increase until reaching a maximum peak value of 1.0 (moment C) during the preictal period. After midnight, the system raised a false alarm (moment B) when it reached a value of 0.7. Despite being a false alarm, we considered this behavior normal in the light of the preictal period assumption: a transitional stage between seizure‐free brain activity and a seizure, able to be captured from an EEG‐background analysis.^14^, ^32^, ^33^ All interviewees accepted that the system raised a false alarm because this brain transition may have started minutes earlier than the interval considered when training the model.

By visualizing patient 93402's seizure #4 (Figure 3c), we identified three distinct patterns of alarms over time: between midnight and 6 am (red circles), around mid‐day (yellow circles), and around 6 pm (green circles). Additionally, in this patient, both testing seizures occurred between midnight and 6 am (seizure #4 at 01:35 and seizure #5 at 06:10), which fit into the red cluster. We presented the following hypothesis: *these false alarm clusters suggest the existence of periods of brain susceptibility to seizures, which may not always lead to seizures*. This rationale is a paradigm shift from prediction to forecasting,^34^ which intuitively appeared during interviews. We observed a Firing Power increase over days in the yellow and green circles, which suggests an effect of medication tapering, as told by one clinician. These hypotheses might explain the occurrence of false positives.

When inspecting Figures 3d,e (seizures #4 and #5 from patient 402), one may hypothesize that around the waking up time, there is a higher risk of seizure (both testing seizures occur at waking up). For these cases, the logistic regression model never raised any alarm (see Table 1).

All these explanations are merely hypotheses, and we must test them. Testing the developed hypothesis is one of the major topics of this paper, which concerns our last lesson.

### 3.3 Interictal and preictal concepts differ between data scientists and clinicians

Clinicians often consider the preictal period a fast spontaneous phenomenon that might start some seconds before the seizure onset. Data scientists' strategy is different: They aim to capture a slow transition from a background state into a seizure, by classifying consecutive windows of EEG as preictal/interictal. These notions of seizure generation are different.

**FIGURE 4:**
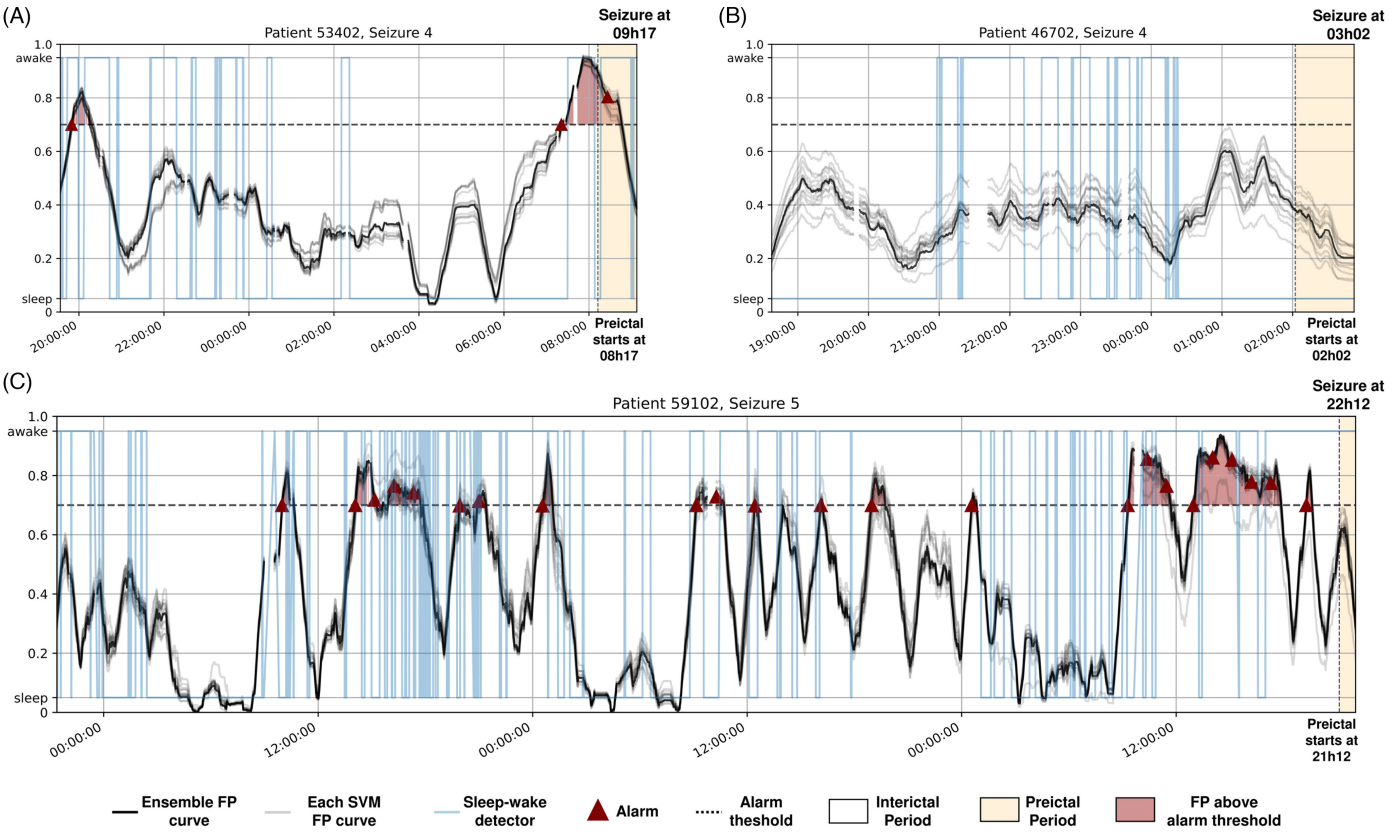
Plot of the ensemble of SVMs' Firing Power for different patients: patient 53 402 seizure #4 (A), patient 46 702 seizure #4 (B), and patient 59 102 seizure #5 (C). Each SVM decision is in gray, and the ensemble voting system is in black.

Figure 4 shows the ensemble of SVMs for patients 53 402, 46 702, and 59 102. These plots show a strong agreement between the ensemble voting (black line) and each SVM's decisions (gray lines). Each SVM model was different as the feature selection and data balance steps were stochastic operators. We asked clinicians and data scientists if this similarity provided trust. The data scientists were satisfied since the SVM output was coherent between each execution. Clinicians were not consensual and tended to refrain from answering due to our definition of preictal and interictal periods.

### 3.4 Making and testing conjectures is the solution to explain a ML model decision when there is no solid physiology grounding

If clinicians cannot explain pre‐seizure mechanisms several minutes (or even hours^32^) before seizure onset, data scientists will not be the ones doing it as they cannot provide a clinician‐comprehensible answer. One clinician stated: “until proven otherwise, everything is an artefact.” This rationale is vital to understand the goal of explainability. For seizure prediction, the goal of explainability is not merely to explain decisions but to develop hypotheses based on physiology mechanisms, which we must test. When we reject the null hypotheses, we gain insight and trust. If they fail, we must review our study assumptions and redesign our methods and explanations, leading to a more complete problem formalization. The loop continues until we can trust our methods.

### 3.5 Making and testing patient‐specific conjectures

As mentioned, patient 8902's logistic regression model achieved high performance using several gamma‐related features, which provoked skepticism since we used scalp EEG, and therefore, it would be difficult to capture gamma activity. We can think of two hypotheses about the gamma features: (i) there might be predictive power in the gamma features related to the GABAergic system and a high‐frequency synchrony increase, or (ii) gamma‐related features might have captured muscle artifacts as patient 8902 presented muscle jerks which resulted from pre‐seizure dynamics. It is important to note that, despite we cleaned our signals, the used preprocessing model had a conservative behavior.^21^ Concerning hypothesis (i), we cannot state and test it because we do not have intracranial EEG recordings of this patient.

Concerning hypothesis (ii), we could ask a clinician to visually inspect the EEG windows where gamma spectral‐band power is significant, but that would be extremely arduous. We could also search for tools that classify signal segments into artifacts, noise, or EEG‐related phenomena, which would also take time. We followed a faster strategy. To understand the gamma‐band contribution, we retrained our model six times using, for each time, one of the following features: spectral‐band power features from either delta, theta, alpha, beta, or gamma bands, signal's time variance, and signal's total band power. We used time variance to understand whether a simple time‐domain measure could also capture similar dynamics.

**FIGURE 5:**
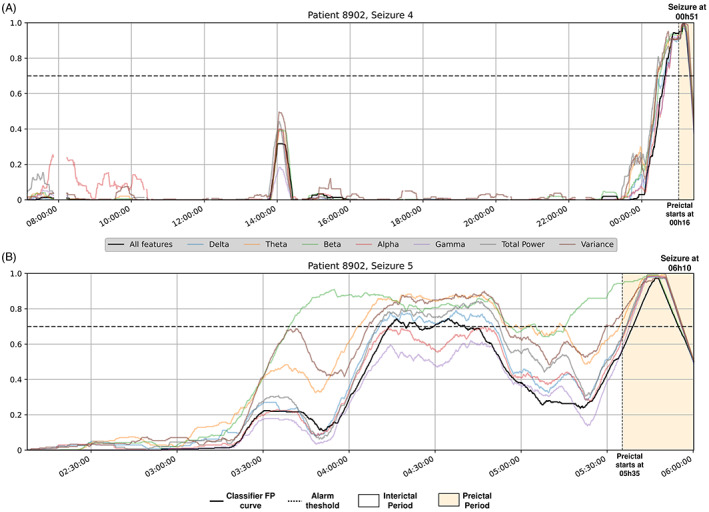
Patient 8902's Firing Power time plots for different logistic regression models for both testing seizures (A and B). The black line represents the original model, the remaining concern logistic regression models trained with only a determined spectral‐band power, total band power, or the signal time variance.

For seizure #4 (Figure 5a), all spectral bands, total spectral power, and time signal variance had a similar discriminatory capacity. With seizure #5 (Figure 5b), despite the majority of the features' models presenting a similar morphology, there were differences. Delta, theta, and total power models would raise more false alarms, while gamma and alpha bands would not raise any. The beta band power and time variance presented differences in their Firing Power dynamics, where the first often exceeded the alarm threshold. With these findings, we mitigated some of the gamma‐band features' skepticism and rejected the muscle‐jerk hypothesis (muscle artifacts typically appear in the beta and gamma bands^35^, ^36^). The same patterns appeared in most spectral bands, total spectra, and within the temporal domain, which suggest a general EEG‐background change^32^, ^33^ due to pre‐seizure dynamics.

For patients 93 902 and 402, we could construct hypotheses related to circadian and sleep–wake cycles, respectively. Nevertheless, we must use more data to test these. As we could not test our hypotheses, we cannot truly state these.

Despite needing more data to confirm these hypotheses, we may include other strategies to speed up this process. The clinicians pointed out their curiosity to understand how our models would behave when patients performed daily activities, such as eating, getting up, scratching their heads, and others. This rationale relates to trying to make our methodology fail: if we could guarantee that our models would not confound these activities with a pre‐seizure state, we would gain more trust. Additionally, when developing methods to detect such activities, we may better understand patients' circadian cycles influence. These strategies can also focus the preprocessing stage.

### 3.6 Making and testing patient‐general conjectures

We also developed and tested other hypotheses from the entire set of patients. By inspecting all patients' Firing Power plots using the logistic regression model, we often found patients that, for at least one seizure, presented one of the following scenarios: (i) the model could not predict a seizure, but we could trust its Firing Power behavior; or (ii) our model's Firing Power behavior was poor, but we could find a sleep–wake transition in the preictal period, suggesting a sleep–wake cycle influence (as patient 402 in Figure 3). We counted 14 and 21 patients for the first and second scenarios. Additionally to patient 93 402, we found four more (59 102, 95 202, 109 502, and 112 802) (Figure 3) where false alarm cycles occurred within similar periods in consecutive days, suggesting a circadian‐cycle influence. See Data S1 for examples to better understand these cases and this paper's Github for all patients' time plots.

Lastly, clinicians asked about a performance bias toward patients whose training and testing seizures occurred around similar times of the day. Thus, we applied a forecasting rationale to the logistic regression models, where high seizure‐risk warnings corresponded to Firing Power values over the alarm threshold. We then compared these forecasting models to a circadian forecasting algorithm that only used circadian information. We only performed this procedure for the logistic regression models for simplicity reasons. For each tested seizure, the circadian algorithm raised high seizure‐risk warnings from 30 minutes before to 30 minutes after each seizure training onset time (see Data S1 for an illustration of this algorithm). By comparing seizure sensitivities and time under warning (TiW), we verified that the EEG‐based models outperformed the circadian forecasting in both metrics. The EEG‐based models obtained a SS = 0.29 and a TiW = 1 h32, while the circadian forecasting obtained a SS = 0.15 and a TiW = 2 h52. Moreover, six EEG‐based patient models presented simultaneously higher sensitivity and lower TiW values. See Data S1 for the full comparison results between the two approaches.

All the counts in this subsection presented statistical significance based on accumulative binomial distribution tests (α = 0.05).^13^, ^14^ We detailed the complete statistical analysis in Data S1.

## 4 DISCUSSION

The obtained prediction performances are far from a clinical application, which might lead to arguing that interpreting a low‐performance model is conceptually flawed. However, by choosing a plethora of performances (high and low), we evaluated the impact of explanations for all case scenarios. Only developing explanations for perfect case scenarios would constitute an incomplete analysis. Sometimes, some explanations justify bad prediction performance, thus still giving the model a certain degree of trust.

Although we used state‐of‐the‐art features to characterize the EEG signal, identify manifestations of the pre‐seizure state, and evaluate the models' Firing Power, our explanations were insufficient to understand brain dynamics of seizure generation. ML explainability might not be the proper tool to understand the EEG, which justifies research efforts on developing specific strategies to make our models fail. Also, Ml research is prone to confirmation bias, easily achieved due to overfitting and overtesting. However, it is difficult for clinicians to reject the developed models when they hold against a systematic conjecture testing. We should be conservative in our explanations and base model development on an iterative refutation algorithm when the underlying physiologic mechanisms are unknown.

We tried to tackle this lack of understanding on the ictogenesis process by showing the EEG to clinicians. During the interviews, we made available the segments marked as relevant. We also showed data samples that the CNNs found relevant in predicting seizures. Clinicians did not find any pattern, which leads us to consider that the CNN classifier did not use a neurologist's rationale. A long‐term EEG monitorization may not lead to a higher understanding of the ictogenesis process, but it can be beneficial to capture factors that may influence a higher susceptibility to seizures (seizure forecasting^34^). Also, note that SHAP and LIME methods explain the driving features behind a model outcome. Because neither of these methods is appropriate for causal inference, their contribution to explaining the presumed precursors of ictogenesis using correlative associations is limited.

For the case of seizure prediction, this work proves that interpretable models may not lead to more convincing explanations. No clinician raised any issue concerning model transparency when presenting the SVM and CNN classifiers, which might be due to the lack of EEG ictogenesis‐related knowledge. Despite the literature heads toward more deep learning,^6^, ^7^, ^37^ we need to stress this finding as some authors advocate against the use of such models due to their black‐box nature^38^ and a still purposely vague legislation.^39^

Explaining seizure prediction models led us to consider each case scenario individually. We considered different hypotheses for each patient and handled their conjecture testing differently. When verifying all patients' time plots, we found typical model behaviors which may be a consequence of epilepsy's clinical heterogeneity. Also, for a significant number of patients, the EEG‐based models outperformed (in both terms of SS and TiW) the circadian forecasting models. Although the used data does not concern real‐life, this finding confirms the added value of developing EEG‐based prediction approaches for some patients.

This work had several limitations. It is hard to provide extensive examples of some conjecture testing strategies, as we used data from presurgical monitoring conditions. Our explanation hypotheses require new testing data and extensive recording periods. Although we have hypothesized possible circadian and sleep–wake cycles' influence, which the literature supports,^34^ we did not have sufficient data to confirm it. It would also be valuable to have access to video EEG and to find more patients presenting a clear EEG‐background transition from a baseline to pre‐seizure activity, as patient 8902. Please, also note that these explanations involved algorithm retraining after visualizing results. Another limitation of this work is the possible bias that involves explanations after visualizing results. Making conjectures, collecting further data, and testing may also solve this issue.

Counterfactual explanations were not relevant. However, they are gaining prominence within many technical, legal, and business circles for ML.^40^ These explanations were not pertinent as patients cannot change their brain dynamics. Counterfactual explanations tend to be more useful when the user can intervene in the decision, such as a bank decision on a loan: *if you had done X, you would have got the loan*. They might be helpful in forecasting when analyzing a large quantity of data and accessing information the patient can control, such as medication, sleeping, and eating.

Our lessons result from GT's iterative and emergent observation and analysis. We encourage authors to perform similar methodologies. Due to our ML seizure prediction background, we recognize an increased difficulty in using this tool.^10^

It is often discussed whether network changes or synchronization may correlate with seizure propensity. These may be crucial to understand the transition between the interictal state and seizures. The used features did not give direct answers to these aspects of brain activity, which is another limitation of our work. We still approached the brain synchronization topic by asking the clinicians about it when they visually analyzed the EEG and CNN decisions. The clinicians could not find any synchronization pattern visually in the EEG or the CNN decisions.

This work may translate to other healthcare applications, particularly EEG‐related, where we do not comprehend the physiological mechanisms that generate an event to be predicted. Other healthcare problems might require a higher degree of transparency as there may be established score models grounded on a clinical rationale.^41^

## 5 CONCLUSION

While comprehensive, this study fell short of proposing a clear and standard methodology that researchers and, primarily, clinicians would apply to black‐box predictive models to make sense of the interworking behind predicting seizures. This is why we preferred to present our findings in terms of lessons. For now, proposing a clear and standard methodology may be hard.

Clinicians do not fully understand EEG pre‐seizure mechanisms occurring several minutes before a seizure; nevertheless, it was possible to provide more or less convincing explanations for certain model decisions without requiring full model transparency. We conclude that, for predicting seizures, explainability is not about simply explaining decisions but improving the developed models, reviewing used assumptions and, thus, gaining trust. Basing model development on an iterative refutation algorithm might promote trust while dealing with a lack of clinical grounding.

For future work, we must repeat this methodology in ultra‐long‐term recordings from real‐life, such as data from Cook et al.^42^ We may find other case scenarios with that data and confirm (or reject) the reported ones. Only a long‐term analysis will tell if these explanation methods remain effective when inspecting months of data. We must also consider interviewing patients^43^, ^44^ to understand their perspectives, relation to devices, and how we can help them deal with the devices' predictions in cases of failure.

## AUTHOR CONTRIBUTIONS

Mauro F. Pinto, César A. Teixeira, and Pedro Martins designed the experiment. Mauro F. Pinto, Fábio Lopes, Adriana Leal, and Ana Oliveira developed code for the seizure prediction pipeline. Mauro F. Pinto and Joana Batista developed code for explaining model decisions. Adriana Leal, Mauro F. Pinto, Fábio Lopes, and António Dourado interpreted and discussed the results concerning a machine learning prediction context. Sulaiman I. Abuhaiba and Francisco Sales interpreted and discussed the results concerning the clinical context. António Dourado reviewed substantially the manuscript. Mauro F. Pinto wrote the manuscript. All authors reviewed the manuscript.

## CONFLICT OF INTEREST STATEMENT

Neither of the authors has any conflict of interest to disclose. We confirm that we have read the Journal's position on issues involved in ethical publication and affirm that this report is consistent with those guidelines.

## Supporting information


Data S1
