# Supplementary material for: The goal of explaining black boxes in EEG seizure prediction is not to explain models' decisions
Source: Epilepsia Open. 2023 Apr 27;8(2):285–97. doi: 10.1002/epi4.12748 (PMC10235568; doi:10.1002/epi4.12748)
Supplement: Supplementary file 1 — Data S1 [file EPI4-8-285-s001.pdf]

Supplementary File
The goal of explaining black boxes in EEG seizure prediction is not to explain models' decisions

Mauro F. Pinto[1,*], Joana Batista[1], Adriana Leal[1], Fábio Lopes[1,2], Ana Oliveira[1], António Dourado[1], Sulaiman I. Abuhaiba[3], Francisco Sales[3], Pedro Martins [1], César A. Teixeira[1]

1 Univ Coimbra, CISUC, Department of Informatics Engineering, Coimbra, Portugal
2 Epilepsy Center, Department Neurosurgery, Medical Center - University of Freiburg, Faculty of Medicine, University of Freiburg, Freiburg, Germany
3 Refractory Epilepsy Reference Centre, Centro Hospitalar e Universitário de Coimbra, EPE, Coimbra, Portugal

* mauropinto@dei.uc.pt

1 Patient Information

Table S 1. Patients' information.

Patient ID	Sex<br>AA	#Seiz. (Train/ Test)	Rec. duration (hours)	Lat.	Surgery decision	Seizure activity pattern	Seizure classification
402	f<br>55	3<br>2	98.14<br>23.07	l, r	i	t, t, t,<br>t, t	FOIA, FBTC, FOIA<br>FBTC, FOIA
8902	f<br>67	3<br>2	124.80<br>21.16	l	n	a, b, a<br>m, a	UC, FOIA, FOIA<br>FOIA, FOIA
11002	m<br>41	3<br>1	91.14<br>10.91	r	s	?, s, a<br>t	UC, FOIA, FOIA<br>FOIA
16202	f<br>46	3<br>4	96.82<br>31.93	l	n	r, ?, r, r<br>r, ?, r	UC, FBTC, UC<br>FOIA, FOIA, FOIA, FOIA
21902	m<br>47	3<br>1	62.56<br>9.42	l	s	t, t, t<br>b	UC, FOIA, FOIA<br>FOIA
23902	m<br>36	3<br>2	65.60<br>30.82	l	s	t, t, t<br>d, t	FOA, FOA, FOA<br>FOA, FOA
26102	m<br>65	3<br>1	58.13<br>21.88	l	i-n	m, t, t<br>t	FOIA, FOIA, FOIA<br>FOIA
30802	m<br>28	3<br>5	82.78<br>58.78	l, r	o	t, t, t<br>t, t, t, t, t	FOA, FOA, FOA<br>FOA, FOA, FOA, FOA, FOA
32702	f<br>62	3<br>2	109.94<br>18.78	l, r	n	t, t, t<br>r, a	FOIA, FOIA, FOIA<br>FOIA, FOIA
45402	f<br>41	3<br>1	68.24<br>18.54	l, r	i	t, t, t<br>t	FOIA, FOIA, FOA<br>FOIA
46702	f<br>15	3<br>2	46.20<br>11.89	r	o	a, a, t<br>b, t	FOA, FOIA, FOIA<br>FBTC, FOIA
50802	m<br>43	3<br>2	158.59<br>33.76	l	i-n	?, a, a<br>t, t, t, t, t	FOIA, UC, UC<br>FOIA, FBTC
52302	f<br>61	3<br>1	70.76<br>6.17	l	i-s	?, ?, d<br>t	UC, FOA, UC<br>UC
53402	m<br>39	3<br>1	65.73<br>12.25	l, r	s	?, ?, ?<br>t	FOA, FOA, FOA<br>FOIA
55202	f<br>17	3<br>5	45.32<br>62.46	l, b	i-n	t, d, t<br>t, t, t, r, r	FOIA, FOIA, FOA<br>UC, UC, FOA, UC, FOIA
56402	m<br>47	3<br>1	111.56<br>19.48	l, r	n	t, ?, ?<br>a	UC, UC, UC<br>FBTC
58602	m<br>32	3<br>3	92.68<br>22.65	l	i-n	r, t, t<br>r, r, t	FOIA, FOIA, FOIA<br>FOIA, FOIA, FOIA
59102	m<br>47	3<br>2	61.90<br>77.60	r	s	?, t, t<br>t, t	FOA, FOIA, FOIA<br>FOIA, FOA
60002	m<br>55	3<br>3	103.27<br>61.07	l, r	s	d, c, t<br>t, d, d	FOIA, FOIA, FOIA<br>UC, FOIA, FOIA
64702	m<br>51	3<br>2	71.45<br>30.15	r	i-s	?, m, t<br>t, t	FOA, FBTC, FBTC<br>FBTC, FBTC
75202	m<br>13	3<br>4	96.59<br>49.52	r	s	t, t, t<br>t, t, ?, t	FOIA, FOIA, UC<br>FOIA, FOIA, FOIA, FOIA
80702	f<br>22	3<br>3	46.29<br>28.62	b	n	b, b, ?<br>c, c, c	FOIA, FOIA, UC<br>FOIA, FBTCB, FOIA
85202	f<br>54	3<br>2	52.01<br>19.61	l	s	m, c, m<br>m, m	FOIA, FOIA, UC<br>UC, UC
93402	m<br>67	3<br>2	92.73<br>50.80	l	n	t, t, t<br>t, t	FBTC, FOIA, FOIA<br>UC, UC
93902	m<br>50	3<br>3	189.32<br>18.49	r	i-n	t, t, d<br>d, d, d	FOA, FOIA, FBTC<br>FOIA, FOIA, UC
94402	f<br>37	3<br>4	112.22<br>28.36	r	o	?, d, b<br>t, ?, b, ?	FOA, UC, FOIA<br>UC, FOA, UC, FOA

Table 1 continued from previous page

95202	f	3	54.24	l	s	b, b, b	FBTC, FOIA, FOIA
	50	4	84.29			m, b, b, t	FOIA, UC, FOIA, UC
96002	m	3	46.05	l, r	s	t, t, t	FOIA, FOIA, FOIA
	58	4	67.38			d, a, t, a	FOIA, UC, FOIA, FOIA
98102	m	3	105.01	l	i-n	?, ?, ?	FOA, UC, UC
	36	2	43.36			?, ?	UC, FBTC
98202	m	3	105.94	r	o	t, a, t	FOIA, FOIA, FOIA
	39	5	45.44			t, t, t, t, t	FBTC, FOIA, FOIA, FOIA, UC
101702	m	3	27.28	l, r	n	t, t, t	FOIA, FOIA, FOIA
	52	2	22.62			r, r	FOIA, FOIA
102202	m	3	54.90	l	n	b, ?, t	FOA, UC, FOIA
	17	4	48.42			?, t, t, t	UC, FOA, FOIA, UC
104602	f	3	82.30	l	o	t, a, t	FOIA, FBTC, FBTC
	17	2	14.47			t, d	FBTC, UC
109502	m	3	72.73	l, r	n	t, t, t	FOIA, FOIA, UC
	50	1	39.11			t	UC
110602	m	3	86.03	r	o	t, t, t	FOIA, FOIA, FOIA
	56	2	24.87			t, t	FOIA, FOA
112802	m	3	68.61	l	n	t, t, t	UC, FOIA, UC
	52	3	103.64			t, t, t	FOIA, FOIA, UC
113902	f	3	57.85	r	o	t, d, t	UC, FOIA, FOIA
	29	3	20.78			t, t, t	FOIA, UC, FOIA
114702	f	3	45.87	r	o	t, t, t	FOIA, FOIA, UC
	22	5	31.67			t, d, t, d, t	FOIA, FOIA, FOIA, FOIA, FOIA
114902	f	3	25.68	l, r	n	s, b, s	FOA, FOIA, FOIA
	16	4	48.73			t, r, a, t	FBTC, UC, FOIA, FOIA
123902	f	3	146.71	l, r	i-n	t, t, t	FBTC, FBTC, FOIA
	25	2	29.21			t, t	FOIA FOA

2 Feature Details

We justify here the use of our features: statistical moments, Hjorth parameters, relative spectral power, wavelet coefficient energy, and spectral edge frequency. We only used linear features. These mathematical techniques use the signal's phase/frequency and amplitude information and comply with the linearity property. When one extracts linear features, assumes the quasi-stationarity of the EEG signal within each time window from the data segmentation step in the signal pre-processing stage.

Statistical Moments

Several studies [1–8] report the use of statistical moments for characterising the amplitude distribution of the EEG time series. The first moment is the mean, the second moment is the variance, the third moment is the skewness, and the fourth moment is the kurtosis. The skewness is zero for symmetric amplitude distributions and non-zero for asymmetric distributions. The kurtosis measures the relative peakedness or flatness of an amplitude distribution [2]. These statistical measures have shown significant changes during the pre-ictal period compared to the inter-ictal state [3, 5]. A decrease in the variance and an increase in the kurtosis were observed in the pre-ictal period when compared with interictal-based data [4, 9].

Hjorth Parameters

Hjorth parameters are three time-domain measures of brain activity that authors have been using in seizure anticipation. These parameters intend to constitute a clinically helpful tool for quantitatively describing the electroencephalogram (EEG) [2]. The activity, mobility, and complexity are measures of mean power, root-mean-squared frequency, and root-mean-square frequency spread, respectively. Authors have reported a significant increase in the mobility and complexity of the EEG during the pre-ictal period [3, 5].

Relative Spectral Power

Different physiological and pathological processes are reflected by activity in different frequency ranges of the power spectrum of the EEG [2]. Relative spectral power band can be calculated by firstly computing the Power Spectral Density (PSD) of the time series within a time window. It is essential to mention that the calculation of the PSD assumes the signal in each window is short enough to be considered quasistationary and long enough to capture the brain's low-frequency activity. Although there are several methods for spectral estimation, the simplest form can be by applying the Fast Fourier Transform (FFT) and then averaging its squared coefficients that belong to the frequency band of interest. A normalised spectral power provides a more robust measure of the fluctuations in a patient's daily life to reveal the pre-ictal state [3]. Authors have reported that brain activity increases or decreases at specific frequency bands during certain events. Before seizure onset, there may be a power transfer from the lower to the higher frequencies [3–5, 10–12]. For example, Mormann et al. [5] showed a decrease in Delta band power, coupled with a relative power decrease in the other sub-bands. Bandarabadi et al. [10] showed that relative combinations of sub-band spectral powers across channel pairs might track gradual changes preceding seizures.

Wavelet Coefficients Energy

Wavelet transform is a time-frequency domain transform that can be an alternative to the FFT as it decomposes the signal in different resolution levels according to different frequency ranges [1, 3, 4]. In other words, wavelets provide a time-variant decomposition adapted to the signal, capturing minor details and sudden changes by providing higher frequency resolution for lower frequencies and higher time resolution to higher frequencies. With the signal decomposed, it is possible to compute several measures using the wavelet coefficients, as in the case of signal energy. By computing the energy of the signals originated by the decomposition, a measure of the energy in different frequency ranges can be achieved [3, 4, 13].

Spectral Edge Frequency

Usually, most EEG signal's power is within the frequency band from 0 Hz up to 40Hz [2]. Spectral edge frequency quantifies the power contained in lower frequencies concerning a given threshold. Spectral edge frequency (SEF) is the measure that indicates the frequency below x per cent of the overall signal power is contained and is frequently used in seizure prediction [1, 3–5]. As studies have found a power transfer from low to high frequencies during the pre-ictal stage, SEF may also be capable of capturing these dynamics.

Decorrelation time

The autocorrelation function is essential in the description of a system's dynamic as it attempts to find repeating patterns as the presence of periodic signals that may be hidden behind noise or to identify the fundamental frequency hidden by its harmonic frequencies. The autocorrelation function compares each point of the time series with other points that are lagged.

When the autocorrelation function has a value close to zero, the time series is uncorrelated, which means there is no similarity between observations in the function of the time lag. When the opposite occurs, its value is close to one. Decorrelation time concerns the first zero-crossing of the autocorrelation function and provides information concerning the typical time scale of the data variability.

Decorrelation time is defined as the first zero-crossing of the autocorrelation function, which can be interpreted as a way to detect time-series stationarity [3]. Authors found that a decrease in the decorrelation time may detect a preictal period [3, 5].

3 Machine Learning Methodologies

We performed a grid search with training seizures during training to find the preictal period (from 30 to 60 minutes). For the logistic regression and the SVMs pipeline, we included the search for the k number of selected features. For the logistic regression, we selected the best k features through the F-test, a filter method that calculates the ratio between variances values [14]. For the SVMs, we used an embedded forest of trees for selecting the best set of features for the following reasons: i) it is computationally light when compared to other embedded methods, and ii) since it is stochastic, it adds another layer of complexity which is desired as we wanted to retrieve explanations from complex methodologies. Finally, in the SVMs pipeline, we also searched the C cost-value.

Table 2 summarises the grid search components of all pipelines.

**Pipeline**	**Preictal Period (30-60 minutes)**	**k features (3,5,7,10,15,20,30)**	**C value (2**c, where c:-10:2:10)**
Log. Reg	x	x	
SVMs	x	x	x
CNNs	x		

Table S 2. Grid search components for each pipeline.

For each set of search parameters (preictal period, number of features, and C value) and each of the three training seizures, we obtained a fitness value by using the geometric mean of sample sensitivity and sample specificity: $\sqrt{sensitivity * specificity}$. We obtained the geometric mean of each seizure as follows: training seizures #1 and #2 and validating #3, training seizures #2 and #3 and validating #1, and training seizures #1 and #3 and validating #2. Then, we averaged the three geometric means to get the fitness value of a given set of search parameters. We then selected the set having the highest fitness value.

In Figures 1, 2, and 3 we show the full details of all pipelines. For the logistic regression feature selection, ANOVA F-test, we used scikit-learn's $SelectKBest(f\_classif, k = n\_features)$ function. For the feature selection in the SVMs pipeline, we used scikit learn's function $RandomForestClassifier$ with max_depth=10, $random\_state$=42, $n\_estimators$=100). We used $linear\_model.LogisticRegression$ and $svm.LinearSVC$ functions from scikit-learn to train our logistic regression and SVM models.

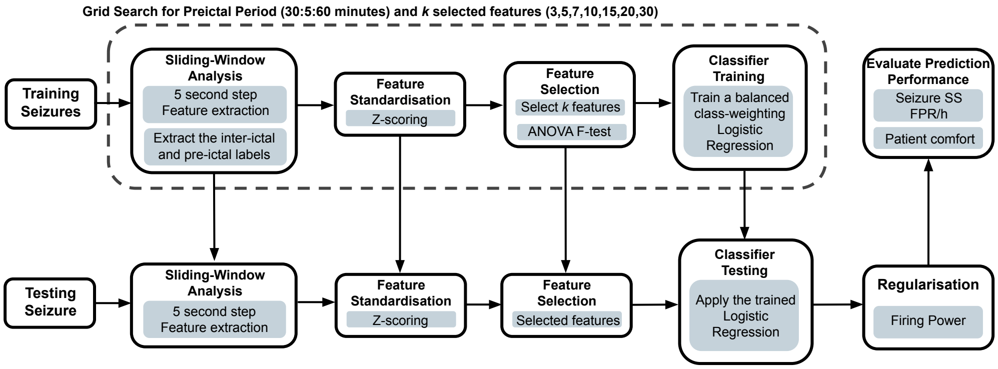

S 1. Logistic regression model pipeline.

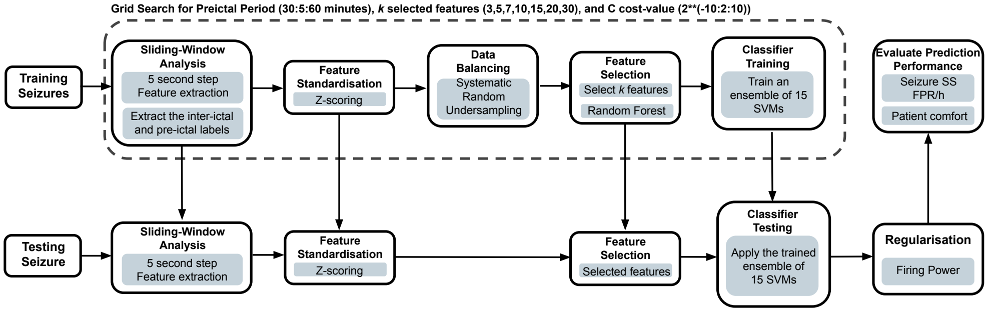

S 2. Ensemble of the SVMs pipeline.

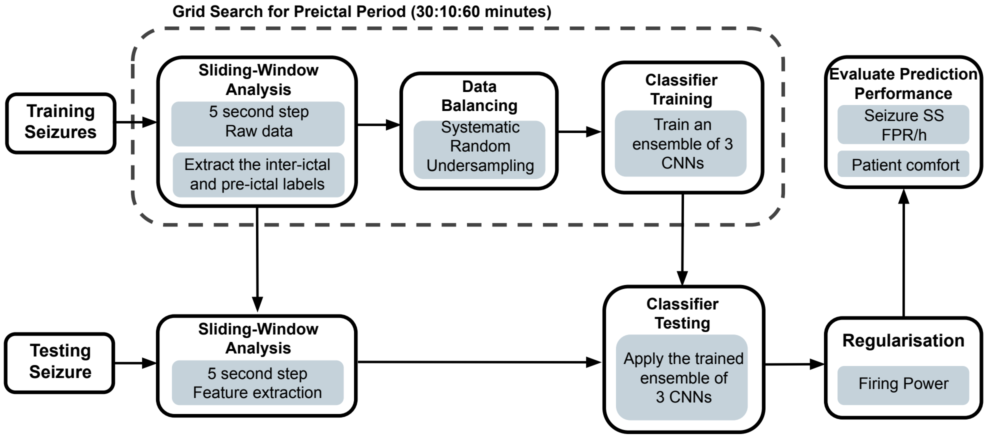

S 3. Ensemble of the CNNs pipeline.

3.1 CNN architecture

We provide here the code to construct our network architecture. We also present a graphical version in Figure 4. We constructed three distinct convolutional parts, each constituted by two convolutional 2D layers. The use of three convolutional components was a common architecture found in the literature [15–17]. We found the filter size and values with an *a priori* grid search procedure. We used convolutional layers with stride instead of max pooling layers as it would help the model learn automatically to reduce dimensionality instead of just performing a fixed operation.

After each convolutional part, we applied a 2D spatial dropout layer, an activation layer, and batch normalisation. Our dropout ratio was high (0.50) to avoid overfitting. We used spatial dropout instead of regular dropout since pixels localisation is essential. In other words, neighbouring pixels correlate with each other as there is a spatial relationship. Therefore, spatial dropout drops entire feature maps instead of just one element, which helps the model to generalise. Batch normalisation layers are for stabilising training by re-centring and re-scaling. We used swish [18] for activation functions instead of Rectifier Linear Units (ReLU) to handle the dying neuron effect (when many ReLU neurons output a zero value, which may mainly happen to a learned negative bias).

We use a global average pooling 2D layer, followed by a dropout layer and a densely-connected layer. Lastly, we used a softmax layer to convert the vector of values to a probability distribution.

```
### CNN Architecture Code ###
```

```
swish_function=tf.keras.activations.swish
nr_filters=32
filter_size=5

input_layer = Input(shape=(1280,19,1))
x=Conv2D(nr_filters,(filter_size,3),(1,1),'same')(input_layer)
x=Conv2D(nr_filters,(filter_size,3),(2,2),'same')(x)
x=SpatialDropout2D(0.5)(x)
x=Activation(swish_function)(x)
x=BatchNormalization()(x)

x=Conv2D(nr_filters*2,(filter_size,3),(1,1),'same')(x)
x=Conv2D(nr_filters*2,(filter_size,3),(2,2),'same')(x)
x=SpatialDropout2D(0.5)(x)
x=Activation(swish_function)(x)
x=BatchNormalization()(x)

x=Conv2D(nr_filters*4,(filter_size,3),(1,1),'same')(x)
x=Conv2D(nr_filters*4,(filter_size,3),(2,2),'same')(x)
x=SpatialDropout2D(0.5)(x)
x=Activation(swish_function)(x)
x=BatchNormalization()(x)

x=GlobalAveragePooling2D()(x)

x=Dropout(0.5)(x)
x=Dense(2)(x)

output_layer = Activation('softmax')(x)

model=Model(input_layer,output_layer)
model.compile(optimizer=Adam(learning_rate=3e-4),
loss='binary_crossentropy',metrics='acc')
```

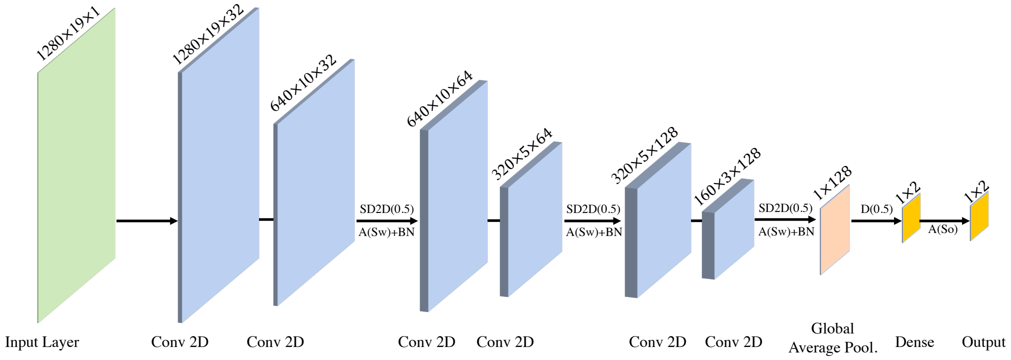

S 4. CNN architecture. Conv2D stands for 2D Convolutional layer, SD2D(0.5) stands for 2D Spatial dropout layer with 0.5 dropout ratio, A(Sw) stands for Activation layer with swish function, BN stands for Batch Normalisation layer, D(0.5) stands for Dropout layer with 0.5 dropout ratio, A(So) stands for Activation layer with softmax function.

4 Other developed explanations and/or more details

In the interviews, we also provided seizure onset times, seizure classification, EEG brain activity pattern and vigilance state at seizure onset. On the feature level, we presented: i) beeswarm summary plots of SHAP Values [19] (Figure 2.b)), which displayed an information-dense summary of how the features impacted the model's output; ii) Partial Dependence Plots (PDPs) [20] and Individual conditional expectation (ICE) [21] plots (Figure 2.c)), which told us the interaction between target response and each input feature; and also iii) logistic regression coefficients, for the logistic regression model (Figure 2.d)). We also discussed the chosen electrodes and features' expected behaviour.

We developed a feature-based explanation inspired by calibration [22] and scatter plots (Figure 2.e)). While a calibration plots the average predicted probability in each bin (x-axis) against the ratio of positive predictions (y-axis), we plotted the feature value (x-axis) against its probability of seizure occurrence (Firing Power value y-axis). In this plot, we also coloured interictal and preictal samples differently. This way, this plot allowed us to visually inspect the classifier behaviour on discriminating individual interictal and preictal windows, and to observe their temporal relation due to the Firing Power values.

For the logistic regression model, we plotted the Firing Power in black. For the 15 SVMs and three CNNs, we plotted all models' Firing Power in grey and the voting decision in black. Then, for moments we considered to be of interest (Figure 2.g)), we plotted the respective points over the Firing Power scatter plot and provided counterfactual explanations [23].

5 Seizure Prediction Results

Patient ID	Preictal Period (minutes)	#Features	SS	FPR/h	Above chance
**402**	**60**	**20**	**0.00**	**0.00**	**0**
**8902**	**35**	**7**	**1.00**	**0.11**	**1**
11002	30	7	0.00	0.78	0
16202	30	30	0.00	0.03	0
21902	50	10	0.00	0.00	0
23902	55	15	0.50	1.35	0
26102	60	30	0.00	0.00	0
30802	60	5	0.20	0.40	0
32702	30	7	0.50	0.06	1
45402	35	20	0.00	0.71	0
46702	40	20	0.00	0.00	0
50802	30	10	0.00	0.28	0
52302	55	30	0.00	1.01	0
53402	50	20	0.00	0.32	0
55202	55	3	0.20	0.55	0
56402	30	10	0.00	0.53	0
58602	30	3	0.00	0.54	0
59102	45	30	0.50	1.05	0
60002	45	7	0.00	0.13	0
64702	50	3	0.00	0.56	0
75202	35	30	0.00	0.04	0
80702	50	30	0.33	0.28	1
85202	30	20	0.00	0.11	0
**93402**	**30**	**3**	**1.00**	**0.50**	**1**
93902	55	5	0.00	0.13	0
94402	55	30	0.00	0.80	0
95202	35	15	0.00	0.37	0
96002	35	3	0.25	0.69	0
98102	35	20	0.00	0.13	0
98202	35	30	0.00	0.02	0
**101702**	**60**	**30**	**0.00**	**0.71**	**0**
102202	60	3	0.00	0.05	0
104602	45	30	0.00	0.40	0
109502	40	20	0.00	2.32	0
110602	50	10	0.50	0.32	1
112802	30	3	0.33	0.75	0
113902	55	30	0.00	0.06	0
114702	45	30	0.00	0.00	0
114902	35	10	0.00	0.00	0
123902	35	3	0.00	0.00	0

Table S 3. Prediction results for the logistic regression model. SS stands for Seizure Sensitivity and FPR/h for False Positive Rate per Hour. Performance above chance was analysed using surrogate analysis. Patients selected for explanations are written in bold.

Patient ID	Preictal Period (minutes)	#Features	SS	FPR/h	Above chance
402	60	30	0.00	0.00	0
8902	30	30	0.00	0.27	0
11002	30	10	0.00	3.93	0
16202	30	20	0.00	0.07	0
21902	45	15	0.00	0.28	0
23902	55	10	0.00	1.34	0
26102	60	30	0.00	0.10	0
30802	60	5	0.40	0.38	0
32702	30	20	0.00	0.12	0
45402	30	15	0.00	1.13	0
**46702**	**60**	**30**	**0.00**	**0.00**	**0**
50802	30	30	0.00	0.17	0
52302	60	3	0.00	1.13	0
**53402**	**60**	**3**	**1.00**	**0.22**	**1**
55202	30	15	0.20	1.11	0
56402	30	15	0.00	3.83	0
58602	30	3	0.00	0.00	0
**59102**	**60**	**15**	**0.00**	**0.52**	**0**
60002	30	30	0.33	0.67	1
64702	50	3	0.00	1.02	0
75202	20	30	0.00	0.11	0
80702	45	30	0.33	0.44	1
85202	60	7	0.50	0.52	1
93402	30	7	0.50	0.77	0
93902	45	3	0.00	0.13	0
94402	30	15	0.00	1.29	0
95202	45	30	0.00	0.12	0
96002	55	20	0.00	1.52	0
98102	60	15	1.00	0.14	1
98202	30	30	0.00	4.14	0
101702	30	7	0.50	1.56	0
102202	60	5	0.00	0.39	0
104602	55	3	0.50	1.49	0
109502	60	7	0.00	0.65	0
110602	45	10	0.50	0.70	1
112802	45	3	0.00	3.01	0
113902	60	3	0.67	1.44	0
114702	45	5	0.00	0.04	0
114902	30	3	0.25	0.21	1
123902	30	3	0.00	0.00	0

Table S 4. Prediction results for the ensemble of SVM models. SS stands for Seizure Sensitivity and FPR/h for False Positive Rate per Hour. Performance above chance was analysed using surrogate analysis. Patients selected for explanations are written in bold.

Patient ID	Preictal Period (minutes)	SS	FPR/h	Above chance
402	60	0.00	0.00	0
**8902**	**60**	**0.50**	**0.00**	**1**
11002	60	0.00	0.00	0

Table 5 continued from previous page

16202	60	0.25	0.08	1
21902	60	0.00	0.00	0
**23902**	**30**	**0.00**	**1.65**	**0**
26102	30	0.00	0.10	0
30802	30	0.20	0.48	0
**32702**	**50**	**0.00**	**0.00**	**0**
45402	40	0.00	0.00	0
46702	30	0.00	0.10	0
50802	40	0.00	0.10	0
52302	60	0.00	0.00	0
53402	50	0.00	0.09	0
55202	60	0.00	0.07	0
56402	40	0.00	0.11	0
58602	40	0.00	0.05	0
59102	50	0.00	0.40	0
60002	50	0.00	0.20	0
64702	40	0.00	0.34	0
75202	30	0.00	0.16	0
80702	40	0.00	0.21	0
85202	30	0.50	0.37	1
93402	30	0.00	0.57	0
93902	45	0.00	0.13	0
94402	40	0.00	0.04	0
95202	30	0.00	0.08	0
96002	60	0.00	0.00	0
98102	40	0.00	0.00	0
98202	50	0.00	1.30	0
101702	30	0.00	0.10	0
102202	60	0.00	0.00	0
104602	40	0.00	0.08	0
109502	30	0.00	0.00	0
110602	50	0.00	0.00	0
112802	50	0.00	0.00	0
113902	30	0.00	0.11	0
114702	50	0.00	0.04	0
114902	30	0.00	0.07	0
123902	30	0.00	0.00	0

Table S 5. Prediction results for the ensemble of CNN models. SS stands for Seizure Sensitivity and FPR/h for False Positive Rate per Hour. Performance above chance was analysed using surrogate analysis. Patients selected for explanations are written in bold.

6 More details about some lessons

Here we provide more details about some of the lessons. We also present another lesson: "Explain your system at different levels". Although important, we decided to move this rule to Supplementary Material as we believe it was intuitive to all readers.

Additional lesson: explain your system at different levels

We advise researchers to divide and order their explanation reasoning into levels according to an increasing granularity or, in other words, from feature and model levels to explaining specific events.

- **Feature level:** show and discuss important features, namely their expected added value, time-window, and correspondent electrode. Also, show each feature's model influence quantitatively. We provided feature influence through beeswarm summary plots of SHAP values, PDP and ICEs, and by analysing the regression coefficients for the logistic regression model. We could also analyse the models' hyperparameters, such as the support vectors from the trained SVM, as suggested during the interviews.
- **Model level:** show how well the model differentiates independent data samples. Graphical explanations are more appealing than only providing fitting metrics and confusion matrixes. We depicted an adapted version of calibration and scatter plots to present the data points' geometric distribution and model discrimination behaviour.
- **Overall system functioning:** provide a visual overview of the system functioning across time and integrate complementary information. Besides providing performance metrics, such as SS and FPR/h, a visual overview provided more information, such as the alarm distribution and the Firing Power over time. We also provided information such as the sleep-wake cycle and time-stamps, which helped to interpret classifier decisions.
- **System functioning over specific moments:** provide deeper explanations concerning potentially decisive moments. We could not inspect explanations about all moments as we dealt with long recordings. People tend only to pay attention and inspect some moments, particularly when models fail: i) false alarms, ii) not predicted seizures, or iii) Firing Power peaks that almost led to false alarms.

Discussing features is important: more details

Spectral bands' relative power was the most discussed feature. Although these were the most appropriate measures to discuss with clinicians, we noticed differences in their conceptualisation between data scientists and clinicians. While clinicians obtain spectral band power by visually inspecting the EEG and looking for spikes, ripples, and others, data scientists use mathematical tools, such as Fourier decomposition. Despite these differences in their computation concerning the clinician interpretation, spectral bands' relative power enables extracting relevant information from long-term recordings.

Interictal and preictal concepts differ between data scientists and clinicians: more details

Understanding these differences is extremely important, as ML model development is based on the provided data. Data scientists deliver data samples to ML algorithms, in

which a sample is usually a 5-second window or mathematical measures (features) computed in that window. Each window is labelled as either preictal or interictal. Then, the ML algorithm trains a model that best discriminates these data samples. Furthermore, other technical aspects also need to be considered. Although the data scientists' preictal period may be more extended than the clinicians' one (a fixed period of usually several minutes until some hours before a seizure), it is still a rare event causing an extreme data imbalance. Also, although this approach is established in literature, it is hard for clinicians to understand that data scientists must define a fixed preictal period for each patient.

7 Interview script

During the presentation to the interviewees, they were allowed to ask questions about any technical aspects or more details. We wanted these interviews to be organic and informal. Thus, we only asked some questions when the interviewees had not spoken about some topics.

In the following list, we show some of the questions asked when the participants did not mention these topics beforehand. We did not approach these topics necessarily in this order.

- What do you think about the presented features?
- Do these features give you enough information?
- What do you think about the sequence of explanations? What do you think about their grouping and order?
- What do you think about the provided explanations? Which ones were more useful? Which ones were less useful?
- Are all these explanations too many?
- What do you think about the explanations about false alarms (patients 8902 and 93402)?
- What do you think about providing circadian and sleep-wake cycle information?
- What do you think about analysing the different 15 SVMs' curves?
- Is there any issue or mistrust related to the models when we go from logistic regression for an ensemble of SVMs or CNNs?
- What are the limitations of the presented explanations?
- Would you like to have any other explanation?

In the end, we always asked the participants if they wanted to say anything else before we finished.

8 General Patient Analysis

8.1 Cases with good Firing Power but failed in prediction

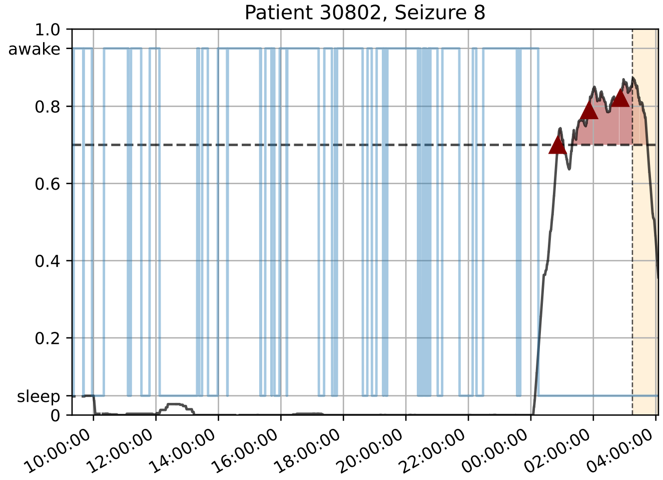

S 5. Seizure #8 of patient 30802. Here we can see that no alarm was raised during the preictal period due to the refractory period of each alarm. When analysing all available hours from that seizure, we can also see that the firing power only started to rise when the patient went to sleep. Thus, all false alarms are close to each other and occur in the last four hours before the seizure onset.

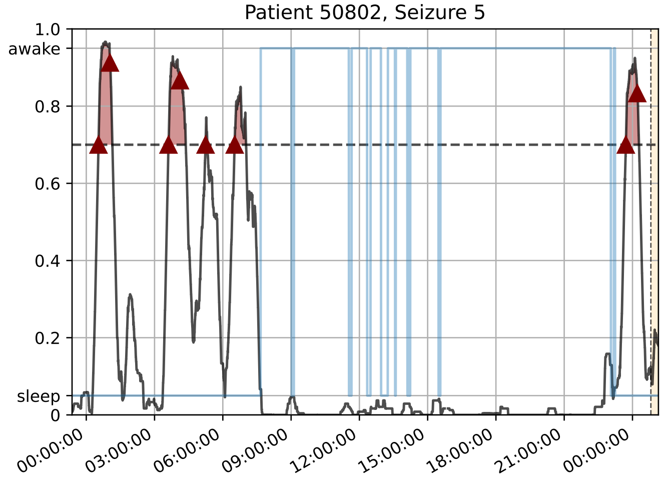

S 6. Seizure #5 of patient 50802. Here we have a clear firing power peak and two false alarms before the preictal period. Nevertheless, we believe in the classifier behaviour as these events occur relatively close to the seizure, and no other alarms occurred before in a near interval (no other alarms until about 17 hours before).

8.2 Circadian-cycle influence in alarms and seizures

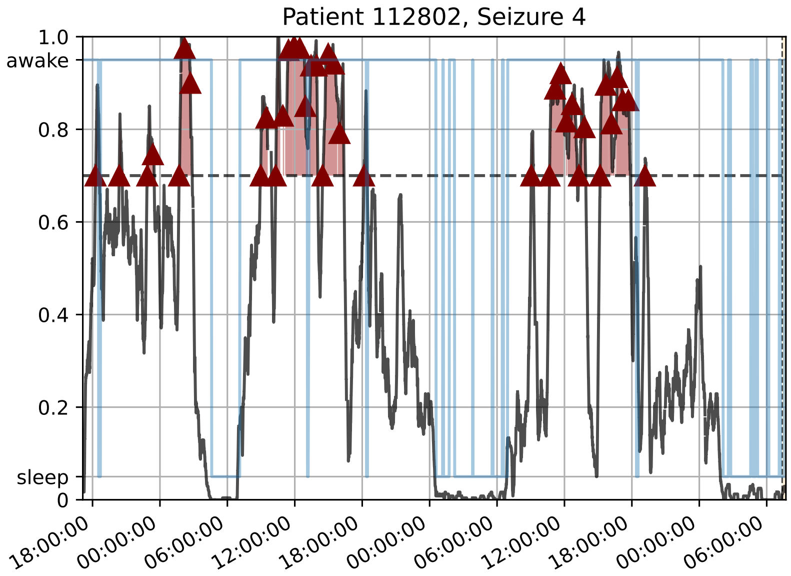

S 7. Seizure #4 of patient 112802. Here we see a false alarm cycle on two consecutive days: from 6am to 6pm. On the third day consecutive day, a seizure occurs after 6am.

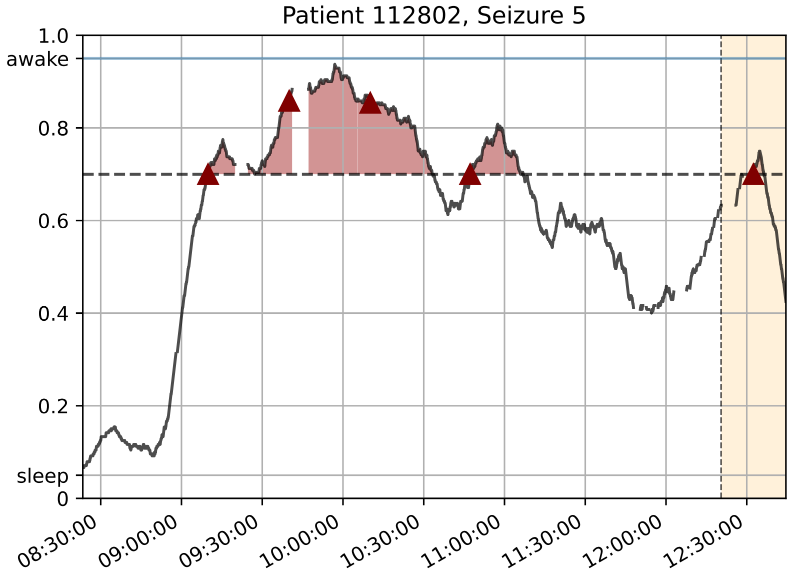

S 8. Seizure #5 of patient 112802. This seizure also occurs within the same cycle lasting from 6am to 6pm.

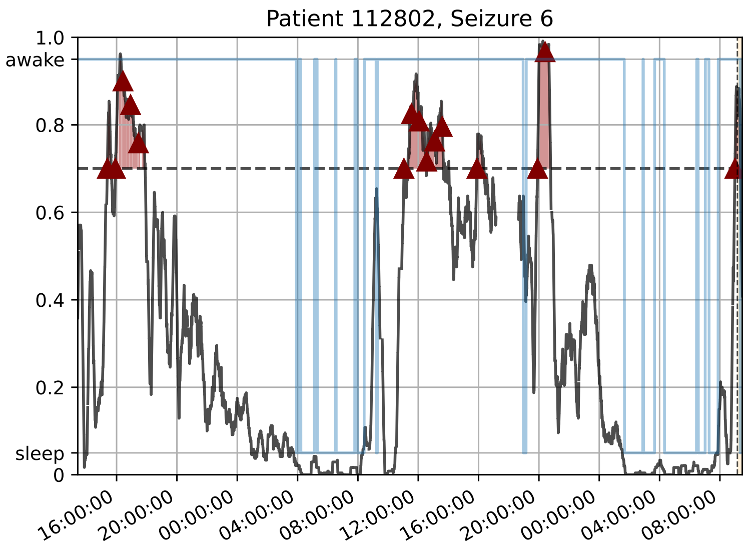

S 9. Seizure #6 of patient 112802. A shorter cycle is observed in this case: from midday to 4pm. Then, the next day, a seizure occurs during the morning. All seizures and the majority of false alarms occurred during the mentioned cycle.

8.3 Sleep-wake transition possible influence

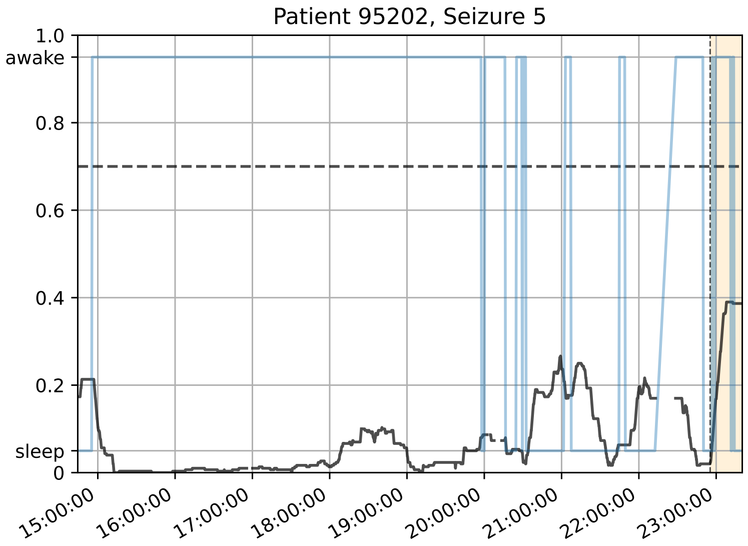

S 10. Seizure #5 of patient 95202. Here we may see the influence of sleep-wake transitions. We can see an influence on these transitions with small firing power peaks. When reaching almost midnight, a seizure occurred.

8.4 Statistical validation

We had to find a way to verify when a certain number of patients having a determined characteristic had statistical significance. In other words, when it was superior to a theoretical chance-level value.

We used the binomial distribution [24] to model the number of successes in a sample of size n drawn with replacement from a population of size N. The binomial distribution may be a good approximation for an N much more extensive than n. The binomial distribution is the basis for the binomial test of statistical significance. We inspired our use of the binomial distribution in previous works [25–27].

Thus, to understand if the number of patients i) whose EEG forecasting was better/equal/worsen than the circadian forecasting or ii) whose circadian cycle influenced alarms and seizures, we used the cumulative binomial distribution (binomcdf) to verify the maximum number of successes given by chance-level. As we do not know the probability of success of these events, we reasonably assumed a p=0.05. As we looked for a significance α=0.05 and assumed a p=0.05, we looked for a number of successes whose probability was under 0.025 as we used a Bonferroni correction [28] (inspired from multiple comparison testing). Although we did not perform multiple comparison tests, we felt we needed to apply this correction since we assumed the p value.

By inspecting Figure 11, we can consider that four successes out of 40 are the maximum regarding chance level. We considered that more than four successes were above chance.

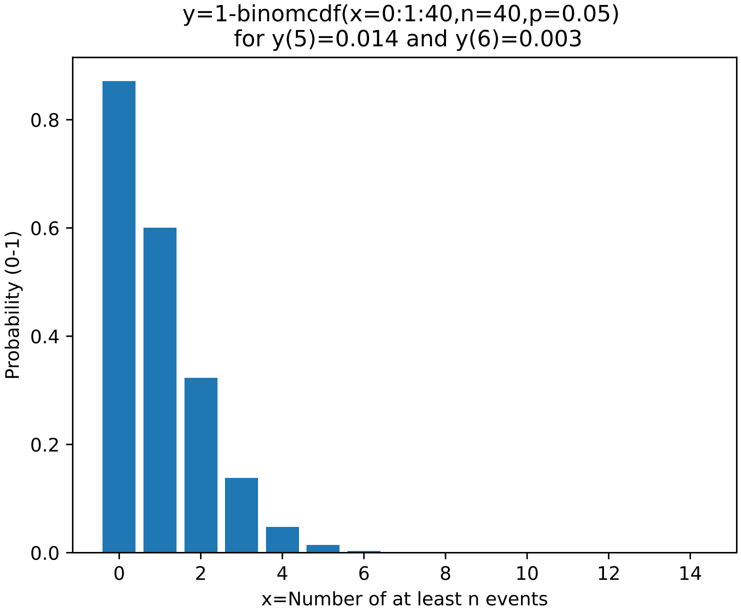

S 11. Graph used to access statistical significance where y=1-binomcdf(x,n,p). Y is the obtained probability (0-1), binomcdf is the cumulative binomial distribution, n is the number of patients (40), p is the probability of success, and x is a vector from 0 to 40 with step 1.

Concerning patients with at least one seizure presenting one determined characteristic (good firing powerbut did not predict the seizure or predictive sleep-wake transitions when the EEG model failed), we made some adaptations to this method. For each patient, we had to account for the possibility of occurring a particular

characteristic in at least one seizure due to luck (by chance). Thus, for each patient, we assumed a p=0.05 and calculated the probability of having success in at least one seizure by chance. The n was the number of tested seizures per patient. We obtained an average interpatient probability of 0.123.

In Figure 12, we provide an example for a patient with three tested seizures, where a probability of success (by chance) is 0.142.

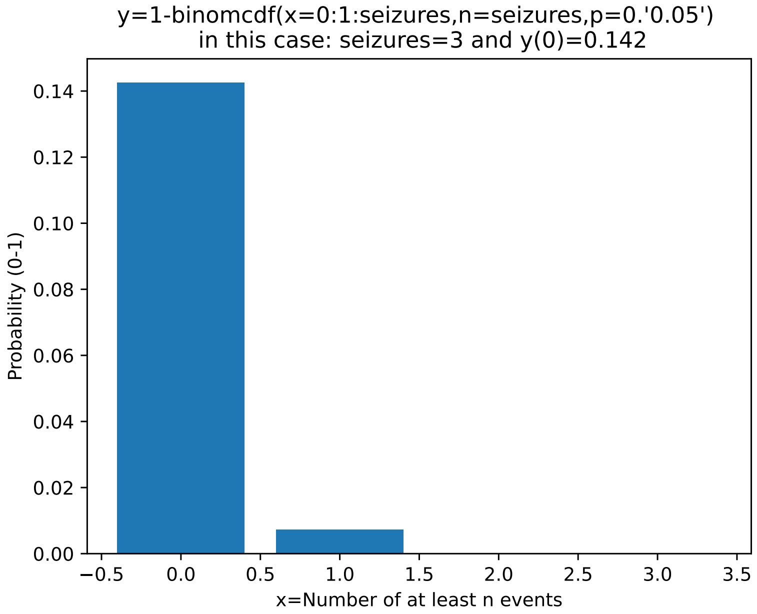

S 12. Graph used to access statistical significance where y=1-binomcdf(x,n,p). Y is the obtained probability (0-1), binomcdf is the cumulative binomial distribution, n is the number of seizures in this patient, p is the probability of success (0.05), and x is a vector from 0 to the number of seizures (3, in this case) with step 1. The obtained probability is y(0)=0.142.

By using a similar rationale to the one in Figure 11, including an additional factor in the Bonferroni correction (probability should be lower than 0.0125), with n=40, x=0:1:40, and p=0.123, we obtained Figure 13. By inspecting this figure, we can consider that nine successes out of 40 are the maximum regarding chance level. We considered that more than nine successes were above chance.

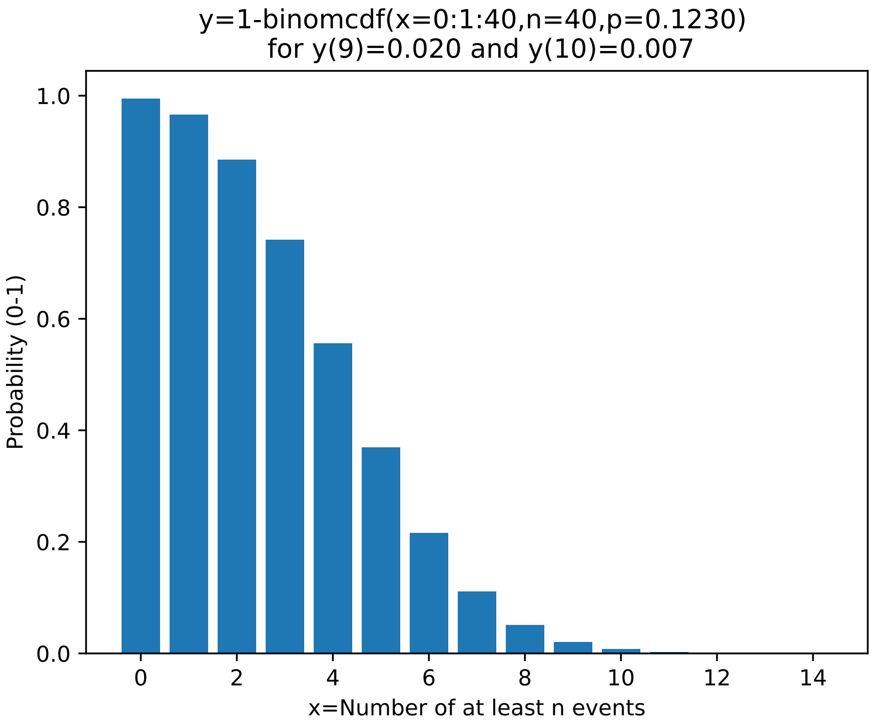

S 13. Graph used to access statistical significance where y=1-binomcdf(x,n,p). Y is the obtained probability (0-1), binomcdf is the cumulative binomial distribution, n is the number of patients (40), p is the probability of success(0.123), and x is a vector from 0 to the number of patients (40) with step 1.

9 Models' comparison with a circadian forecasting algorithm

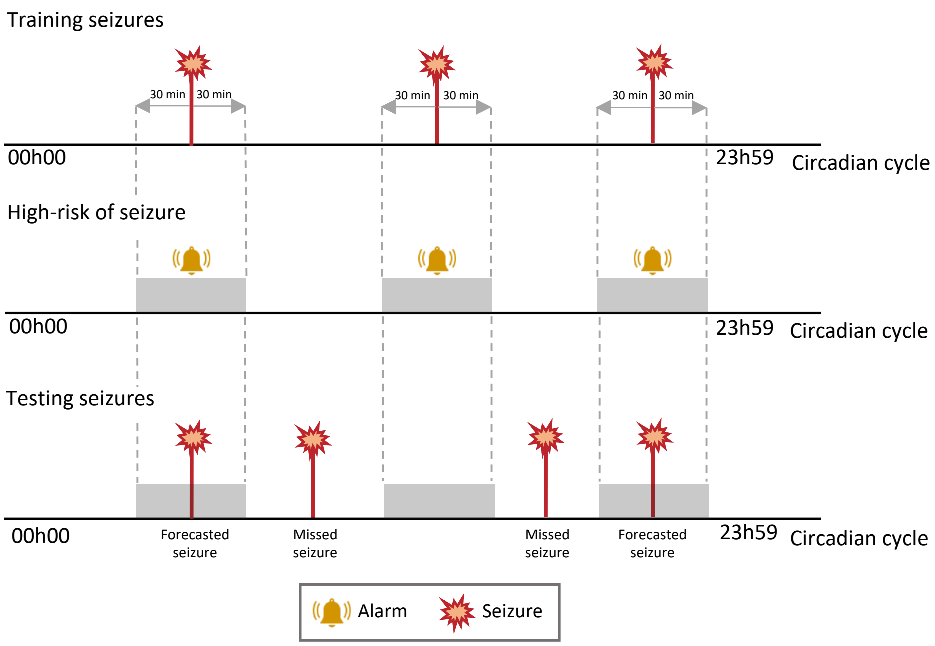

S 14. An example of the circadian forecasting algorithm.

Table S 6. The comparison of the EEG-based forecasting algorithm with a circadian forecasting algorithm.

	Seizure sensitivity					Time under warning (TiW)		
**Patient**	**Circadian model**	**EEG model**	**Circadian > EEG**	**Circadian = EEG**	**EEG > Circadian**	**Circadian model**	**EEG model**	**EEG time <Circadian**
402	0.00	0.00	0	1	0	3h00	0h00	1
8902	0.50	1.00	0	0	1	2h47	1h16	1
11002	0.00	0.00	0	1	0	3h00	1h46	1
16202	0.00	0.00	0	1	0	2h31	0h19	1
21902	0.00	0.00	0	1	0	3h00	0h00	1
23902	0.00	1.00	0	0	1	2h59	11h22	0
26102	0.00	0.00	0	1	0	2h40	0h00	1
30802	0.00	0.80	0	0	1	2h18	14h45	0
32702	0.00	0.50	0	0	1	2h09	0h25	1
45402	1.00	0.00	1	0	0	3h00	1h45	1
46702	0.50	0.00	1	0	0	3h00	0h00	1
50802	0.00	0.00	0	1	0	3h00	2h31	1
52302	0.00	0.00	0	1	0	3h00	2h36	1
53402	0.00	1.00	0	0	1	3h00	1h38	1
55202	0.40	0.80	0	0	1	3h00	16h26	0
56402	0.00	0.00	0	1	0	2h56	2h10	1
58602	0.33	0.00	1	0	0	3h00	2h47	1
59102	0.00	1.00	0	0	1	3h00	24h08	0
60002	0.00	0.00	0	1	0	2h22	3h29	0
64702	0.00	0.50	0	0	1	3h00	6h41	0
75202	0.00	0.00	0	1	0	3h00	0h43	1
80702	0.33	0.33	0	1	0	3h00	3h24	0
85202	0.00	0.50	0	0	1	3h00	0h31	1
93402	0.00	1.00	0	0	1	3h00	8h06	0
93902	0.00	0.33	0	0	1	3h00	0h55	1
94402	0.00	0.25	0	0	1	3h00	5h54	0
95202	0.00	0.25	0	0	1	2h07	8h18	0
96002	0.00	0.25	0	0	1	2h36	14h43	0
98102	0.00	0.50	0	0	1	3h00	1h16	1
98202	0.00	0.00	0	1	0	3h00	0h14	1
101702	0.50	0.50	0	1	0	3h00	7h28	0
102202	0.50	0.00	1	0	0	3h00	1h09	1
104602	0.00	0.00	0	1	0	3h00	1h47	1
109502	1.00	0.00	1	0	0	3h00	16h38	0
110602	0.00	0.50	0	0	1	3h00	4h15	0
112802	0.00	0.66	0	0	1	3h00	21h35	0
113902	0.33	0.00	1	0	0	3h00	0h46	1
114702	0.40	0.00	1	0	0	3h00	0h00	1
114902	0.00	0.00	0	1	0	3h00	1h46	1
123902	0.00	0.00	0	1	0	2h14	0h00	1
	**Average SS**		**Relative frequency**			**Average TiW**		**Relative frequency**
	0.15	0.29	0.18	0.40	0.420	02h52	01h32	0.62

References

1. Direito B, Teixeira CA, Sales F, Castelo-Branco M, Dourado A. A Realistic Seizure Prediction Study Based on Multiclass SVM. Int J Neural Syst. 2017;27(03):1750006. doi:10.1142/S012906571750006X.

2. Mormann F, Andrzejak RG, Elger CE, Lehnertz K. Seizure Prediction: The Long and Winding Road. Brain. 2007;130(2):314–333. doi:10.1093/brain/awl241.

3. Rasekhi J, Mollaei MRK, Bandarabadi M, Teixeira CA, Dourado A. Preprocessing Effects of 22 Linear Univariate Features on the Performance of Seizure Prediction Methods. Journal of Neuroscience Methods. 2013;217(1-2):9–16. doi:10.1016/j.jneumeth.2013.03.019.

4. Teixeira C, Direito B, Bandarabadi M, Le Van Quyen M, Valderrama M, Schelter B, et al. Epileptic Seizure Predictors Based on Computational Intelligence Techniques: A Comparative Study with 278 Patients. Computer Methods and Programs in Biomedicine. 2014;114(3):324–336. doi:10.1016/j.cmpb.2014.02.007.

5. Mormann F, Kreuz T, Rieke C, Andrzejak RG, Kraskov A, David P, et al. On the predictability of epileptic seizures. Clinical neurophysiology. 2005;116(3):569–587.

6. Moghim N, Corne DW. Predicting epileptic seizures in advance. PloS one. 2014;9(6):e99334.

7. Bou Assi E, Nguyen DK, Rihana S, Sawan M. Towards Accurate Prediction of Epileptic Seizures: A Review. Biomedical Signal Processing and Control. 2017;34:144–157. doi:10.1016/j.bspc.2017.02.001.

8. Gadhoumi K, Lina JM, Mormann F, Gotman J. Seizure Prediction for Therapeutic Devices: A Review. Journal of Neuroscience Methods. 2016;260(029):270–282. doi:10.1016/j.jneumeth.2015.06.010.

9. Aarabi A, Fazel-Rezai R, Aghakhani Y. EEG seizure prediction: measures and challenges. In: 2009 Annual International Conference of the IEEE Engineering in Medicine and Biology Society. IEEE; 2009. p. 1864–1867.

10. Bandarabadi M, Teixeira CA, Rasekhi J, Dourado A. Epileptic seizure prediction using relative spectral power features. Clinical Neurophysiology. 2015;126(2):237–248.

11. Howbert JJ, Patterson EE, Stead SM, Brinkmann B, Vasoli V, Crepeau D, et al. Forecasting seizures in dogs with naturally occurring epilepsy. PloS one. 2014;9(1):e81920.

12. Park Y, Luo L, Parhi KK, Netoff T. Seizure prediction with spectral power of EEG using cost-sensitive support vector machines. Epilepsia. 2011;52(10):1761–1770.

13. Gadhoumi K, Lina JM, Gotman J. Seizure prediction in patients with mesial temporal lobe epilepsy using EEG measures of state similarity. Clinical Neurophysiology. 2013;124(9):1745–1754.

14. Venkatesh B, Anuradha J. A review of feature selection and its methods. Cybernetics and Information Technologies. 2019;19(1):3–26.

15. Usman SM, Khalid S, Aslam MH. Epileptic seizures prediction using deep learning techniques. Ieee Access. 2020;8:39998–40007.

16. Usman SM, Khalid S, Bashir S. A deep learning based ensemble learning method for epileptic seizure prediction. Computers in Biology and Medicine. 2021;136:104710.

17. Daoud H, Bayoumi MA. Efficient epileptic seizure prediction based on deep learning. IEEE transactions on biomedical circuits and systems. 2019;13(5):804–813.

18. Ramachandran P, Zoph B, Le QV. Searching for activation functions. arXiv preprint arXiv:171005941. 2017;.

19. Lundberg SM, Lee SI. A unified approach to interpreting model predictions. In: Advances in neural information processing systems; 2017. p. 4765–4774.

20. Friedman JH. Greedy function approximation: a gradient boosting machine. Annals of statistics. 2001; p. 1189–1232.

21. Goldstein A, Kapelner A, Bleich J, Pitkin E. Peeking inside the black box: Visualizing statistical learning with plots of individual conditional expectation. Journal of Computational and Graphical Statistics. 2015;24(1):44–65.

22. Vuk M, Curk T. ROC curve, lift chart and calibration plot. Advances in methodology and Statistics. 2006;3(1):89–108.

23. Wachter S, Mittelstadt B, Russell C. Counterfactual explanations without opening the black box: Automated decisions and the GDPR. Harv JL & Tech. 2017;31:841.

24. Edwards A. The meaning of binomial distribution. Nature. 1960;186(4730):1074–1074.

25. Alvarado-Rojas C, Valderrama M, Fouad-Ahmed A, Feldwisch-Drentrup H, Ihle M, Teixeira C, et al. Slow Modulations of High-Frequency Activity (40-140 Hz) Discriminate Pre-Ictal Changes in Human Focal Epilepsy. Scientific reports. 2014;4:4545.

26. Pinto M, Leal A, Lopes F, Dourado A, Martins P, Teixeira CA, et al. A personalized and evolutionary algorithm for interpretable EEG epilepsy seizure prediction. Scientific reports. 2021;11(1):1–12.

27. Pinto M, Coelho T, Leal A, Lopes F, Dourado A, Martins P, et al. Interpretable EEG seizure prediction using a multiobjective evolutionary algorithm. Scientific reports. 2022;12(1):1–15.

28. Weisstein EW. Bonferroni correction. https://mathworld wolfram com/. 2004;.
